# PCGM-Net: Policy-Conditioned Local Generative Masking for Privacy-Preserving Wi-Fi CSI Sensing

**DOI:** 10.3390/s26165096

**Published:** 2026-08-11

**Authors:** Wei Zhang, Yifu Zeng, Qinglong Tian, Qingmiao Xiong, Honglei Chai, Yingchun Yan, Yuxi Xiao

**Affiliations:** College of Computer Science and Engineering, Changsha University, Changsha 410022, China; chinatql@ccsu.edu.cn (Q.T.); qmx_ccsu@126.com (Q.X.); 13331723838@163.com (H.C.); 15274512362@163.com (Y.Y.); xiyuxiaod@outlook.com (Y.X.)

**Keywords:** Wi-Fi sensing, channel state information, semantic privacy, local generative masking, policy-conditioned protection, trusted-boundary release

## Abstract

Wireless channel state information (CSI) enables device-free industrial safety monitoring, but the same representation can expose worker identity and sensitive locations. Existing CSI privacy methods typically protect fixed semantic targets or perturb the entire representation, providing limited control over what is protected and where modification occurs. This paper proposes PCGM-Net, a policy-conditionedlocal generative masking framework for selective CSI semantic release. To the best of our knowledge, it is the first representation-level Wi-Fi CSI framework to jointly combine explicit semantic privacy policies, a learned position-wise soft mask over the time–subcarrier plane, bounded residual transformation, and trusted retention of the source CSI. The mask determines where intervention is applied, whereas the residual patch determines how the selected regions are transformed. A single model supports identity-only, location-only, and joint protection. Under a test-set-isolated protocol, raw CSI yielded identity and location accuracies of 97.95% and 100.00%, respectively. Across three independently trained protection models selected using validation data only, joint protection retained 74.79±0.96% activity accuracy while reducing identity and location accuracies for the validation-selected evaluator to 6.09±2.40% and 0.29±0.14%. Removing the privacy-margin objective restored identity and location accuracies to 98.55% and 100.00%, confirming that suppression arose from targeted semantic optimization rather than incidental signal corruption. An independent temporal bidirectional gated recurrent unit (BiGRU) model recovered 94.09±1.16% activity accuracy after protected-domain adaptation, and the complete pipeline required 2.18 ms mean graphics processing unit (GPU) latency. PCGM-Net therefore provides low-latency, policy-selective inference-time semantic shielding under a bounded, evaluator-dependent threat model rather than irreversible anonymization.

## 1. Introduction

Industrial safety monitoring increasingly depends on sensor networks, artificial intelligence, and data-driven decision systems. Machine-learning applications in this domain span both worker-centered monitoring and equipment-centered reliability management. For example, top-view vision models have been developed for real-time detection of ground personnel in complex industrial sites, while deep recurrent graph-convolutional architectures have been used for sensor-fault detection, isolation, and accommodation in digital twins [1,2]. Within the Industrial Internet of Things (IIoT), these heterogeneous monitoring and control use cases impose different requirements for latency, reliability, continuity, and edge processing [3]. The safety-monitoring scenario considered here focuses on worker-centered, device-free observation and requires low-latency inference, tolerance to incomplete or degraded CSI, and protection before external storage or downstream transmission. In high-risk workplaces, the timely recognition of falls, abnormal worker movements, restricted-area entry, and other safety-relevant events can improve situational awareness and support earlier intervention. Vision-based systems remain effective in many settings, but their performance and acceptability may be constrained by occlusion, illumination variation, dust, limited field of view, and visual-privacy concerns. Wi-Fi channel state information (CSI) provides a complementary device-free sensing modality because human presence and motion alter multipath propagation without requiring wearable devices or direct line-of-sight observation. CSI has consequently been investigated for activity recognition, human identification, localization, gesture sensing, object measurement, and related tasks [4,5,6,7].

The semantic richness that makes CSI useful also creates a selective-disclosure problem. In representative industrial applications, activity recognition can support incident detection, restricted-zone monitoring, evacuation awareness, and worker-state assessment, whereas identity and room or work-zone information may reveal personnel assignments, restricted-area presence, or behavioral patterns beyond the needs of the authorized service. A CSI sample acquired to detect a fall or departure from a monitored area may simultaneously reveal who generated the motion and where it occurred. The same representation can therefore contain information required by an authorized safety service and information that is unnecessary or sensitive for that service. Industrial wireless sensing consequently requires more than indiscriminate signal concealment: activity information may need to remain accessible, whereas identity, location, or both may need to be withheld according to the active privacy policy. The problem addressed in this work is thus selective semantic protection of an already acquired CSI representation.

Existing Wi-Fi sensing privacy defenses operate at several layers. Representation-level methods generate adversarial or transformed CSI to reduce the recognition capability of a predefined classifier [8,9]. These studies established that semantics embedded in CSI can be selectively disrupted and that a generated representation can retain some useful information while suppressing another target. However, the transformation is generally applied to the representation as a whole, and the privacy target is embedded in the trained transformation rather than supplied as an explicit runtime condition. Such methods do not directly learn which local time–subcarrier positions should receive privacy-oriented modification.

A second line of work intervenes at the transmission, channel-estimation, or physical-sensing layer. The OpenWiFi CSI fuzzer imposes an artificial channel response at the transmitter so that an authorized receiver can recover the actual response while an unauthorized observer obtains a distorted CSI measurement [10]. More recent CSI-fuzzing and frame-obfuscation approaches modify training fields, virtual channel responses, or complete wireless frames to prevent unauthorized sensing [11,12]. Related systems encrypt multi-antenna measurements or employ external backscatter devices [13,14]. These approaches protect the sensing channel effectively, but they generally require control over transmitters, firmware, packet pilots, channel filters, dedicated hardware, or authorized de-obfuscation procedures. They do not directly address selective release of multiple coexisting semantics from CSI that has already been acquired by a trusted sensing system.

Model- and data-level defenses provide another form of privacy control. Poison2Cure, for example, manipulates user-provided calibration data so that private activities become difficult to recognize while regular activities remain detectable [15]. Such methods offer semantic protection without modifying communication hardware, but their protection targets are tied to activity classes and to the calibration or training process. They do not generate a policy-conditioned protected view of an acquired CSI sample, nor do they identify local regions within that sample where privacy-oriented intervention should be applied. More broadly, recent surveys show that wireless-sensing privacy can be addressed at the transmitted signal, estimated channel, learned representation, model-training process, and application-output layers [16,17]; nevertheless, no single representation-level mechanism jointly addresses local intervention, runtime semantic-policy switching, and trusted separation between source CSI and the representation released downstream.

This gap is important because activity-, identity-, and location-related evidence need not contribute uniformly across the CSI map. Although these semantics may overlap, different temporal intervals and subcarrier regions can contribute differently to downstream inference. A transformation applied indiscriminately to the complete representation may suppress private semantics, but it may also modify regions that remain useful for authorized activity recognition. A more selective mechanism should therefore determine where privacy-oriented intervention is needed and should separate this allocation decision from how the selected regions are transformed.

The first challenge is consequently local intervention. The objective is not merely to generate a stronger perturbation, but to allocate modification over the time–subcarrier plane. The location of the intervention and its signed magnitude should be represented by different model components. In this paper, local refers to spatially varying gating relative to an ungated full-map transformation. It does not imply a binary, extremely sparse, or uniquely interpretable physical segmentation of CSI.

The second challenge is policy flexibility. Privacy requirements vary across downstream services and operating contexts. One service may require identity protection while retaining location information, another may require location protection while retaining identity information for an authorized operator, and a third may require both attributes to be suppressed. Training and maintaining a separate transformation model for each policy weakens operational flexibility. The protection target should therefore be supplied explicitly at runtime so that one model can generate different protected representations from the same acquired CSI.

The third challenge concerns the data-flow boundary. Protection of a released representation must be distinguished from preservation of its source. Modifying what is exposed downstream does not require the raw CSI to be overwritten, reconstructed, or permanently altered. A practical architecture should retain raw CSI inside a trusted receiver-side or edge-processing boundary and release only a separately generated, policy-conditioned representation. In this work, source-preserving refers to trusted retention of the original CSI, not to numerical or physical equivalence between the raw and released representations.

The fourth challenge is empirical validity. Representation-level protection can appear effective merely because it induces distribution shift for one fixed classifier. A credible evaluation must therefore isolate the final test set from all model and coefficient selection, use multiple independently trained protection models, examine clean and degraded CSI, and identify the distinct roles of the architectural and objective components. It should also test whether an independent authorized model can recover useful activity semantics after protected-domain adaptation, whether labeled protected-domain retraining can relearn private semantics, whether safety-critical false negatives remain acceptable, how the representation changes structurally, and whether the computational overhead is compatible with real-time processing on the evaluated platforms.

To address these challenges, we propose PCGM-Net, a source-preserving, policy-conditioned generative masking framework for selective CSI semantic protection. Its central innovation is a learned local gating mechanism over the time–subcarrier plane. Given CSI and an explicit privacy policy for identity and location, PCGM-Net predicts a position-wise soft mask and a bounded residual patch. The mask determines where intervention is applied, whereas the residual patch determines how the selected positions are transformed. Their gated product generates a separate protected representation while the original CSI remains unchanged inside the trusted boundary.

A single PCGM-Net supports identity-only, location-only, and joint identity–location protection. Its optimization objective preserves authorized activity information, suppresses semantics selected by the active policy, discourages unnecessary suppression of a private semantic not selected by the policy, regularizes mask extent, and limits representation distortion. The aim is therefore not indiscriminate recognition failure, but controlled semantic accessibility under an explicit release policy.

To the best of our knowledge, PCGM-Net is the first representation-level Wi-Fi CSI protection framework to combine a learned local mask, explicit runtime policies for identity and location, bounded residual modification, and trusted retention of the source CSI. This claim is deliberately limited to the combined design. It does not imply that adversarial CSI transformation, semantic privacy protection, wireless obfuscation, or masking in wireless security is individually unprecedented.

**Figure 1:**
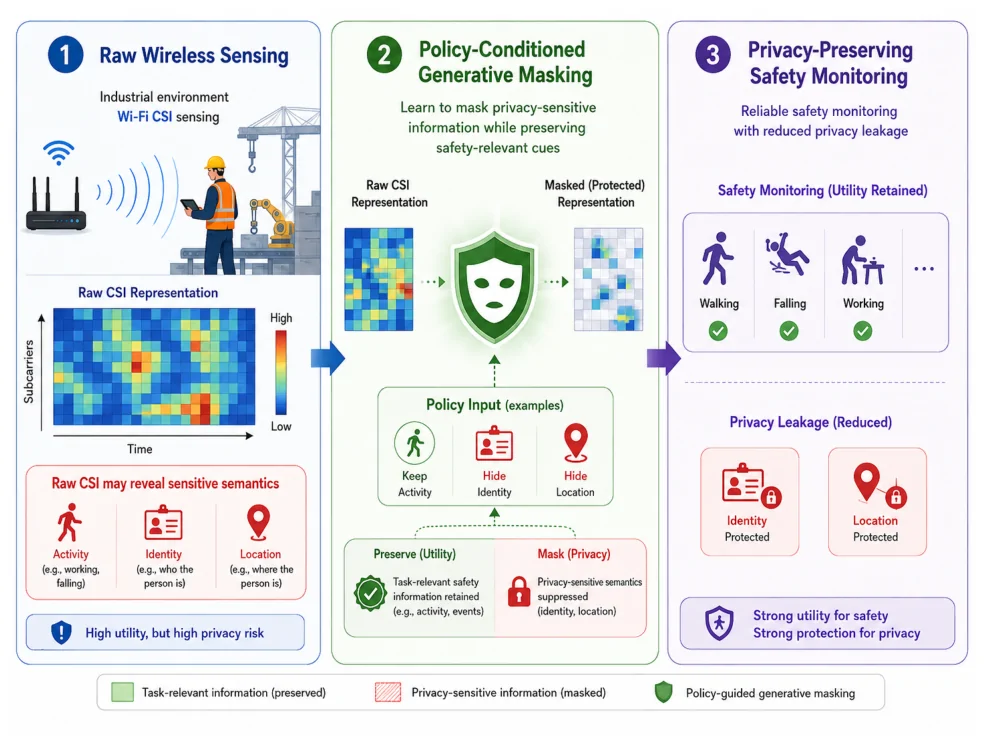
Conceptual workflow of policy-conditioned selective CSI release. Raw CSI remains inside the trusted boundary, and only the policy-conditioned protected representation is released downstream.

Figure 1 illustrates the system boundary and information flow. Raw CSI remains inside the trusted sensing boundary, while the runtime policy specifies whether identity, location, or both should be protected. PCGM-Net uses a position-wise soft mask to allocate intervention and a bounded residual patch to determine the applied modification; only the resulting protected representation is released downstream. Source preservation therefore refers to retaining the original CSI inside the trusted system rather than leaving the released representation unchanged. The primary threat is semantic inference from the released view without access to the raw-CSI store. Because labeled protected-domain retraining may recover private semantics, PCGM-Net is not presented as cryptographic protection or irreversible anonymization; adaptive attackers delimit this boundary, while independent activity models evaluate recoverable authorized utility.

The main contributions are as follows:

1. Local generative masking for CSI semantic protection. We introduce a representation-level protection mechanism that separates intervention allocation from transformation magnitude. A position-wise soft mask controls where modification is applied over the time–subcarrier plane, while a bounded residual patch controls how the selected regions are transformed. Relative to an ungated full-map residual, the learned mask restricts the spatial extent of intervention without requiring the raw CSI to be overwritten.
2. Policy-conditioned multi-semantic protection. We formulate selective CSI protection as an explicit policy-conditioned learning problem. One PCGM-Net supports identity-only, location-only, and joint protection at runtime. The objective suppresses policy-selected semantics while preserving authorized activity information and discouraging unnecessary suppression of a private semantic not selected by the current policy.
3. Trusted-boundary semantic release. We define a system-level protocol that separates trusted source retention from protected downstream disclosure. Raw CSI remains inside the sensing or edge-processing boundary, whereas external storage and analytics receive only a separately generated protected representation. The framework is therefore non-destructive with respect to the source data, although the released view is intentionally modified.
4. Test-set-isolated and boundary-oriented validation. We evaluate the framework using training-only normalization, validation-only checkpoint selection, an untouched test partition, and three independent model seeds. The analysis combines a nine-configuration ablation analysis, hyperparameter sensitivity, learned full-map baselines, degradation and safety-critical evaluation, structural diagnostics, architecture-diverse post-protection attackers, independent activity-model adaptation, and profiling on graphics processing unit and central processing unit platforms. This evidence distinguishes inference-time semantic shielding from irreversible anonymization and identifies the conditions under which authorized utility can be recovered.

The remainder of the paper is organized as follows. Section 2 reviews CSI semantic inference and wireless-sensing privacy protection. Section 3 formalizes the selective-release problem and threat model. Section 4 presents PCGM-Net and its optimization objective. Section 5 integrates the experimental protocol, empirical results, mechanism analysis, adaptive-boundary evaluation, and deployment implications. Section 6 concludes the paper and summarizes the remaining limitations.

## 2. Related Work

### 2.1. Multi-Semantic Inference and Robustness in Wireless Sensing

Wi-Fi channel state information (CSI) records time-varying and frequency-selective channel responses produced by human motion, body-dependent scattering, and environmental multipath. This structure has supported both physically informed and data-driven sensing. Dynamic Fresnel-zone modeling explains how human movement perturbs propagation paths and generates activity-dependent CSI patterns [18]. Deep architectures further extract semantic information from amplitude and phase representations: WiPhase reconstructs and fuses phase features for activity recognition, vision-transformer models capture long-range dependencies over time and subcarriers, and self-supervised learning exploits latent CSI structure with reduced label dependence [19,20,21]. Beyond Wi-Fi, millimeter-wave systems have demonstrated facial-expression, stress, and human-state inference from radio reflections [22,23,24]. Collectively, these studies show that wireless measurements are not task-specific signals: one representation may contain behavioral, person-dependent, and spatial semantics simultaneously.

The same literature also shows that semantic recoverability is sensitive to environment, device placement, link geometry, hardware, and data availability. Meta-learning and domain adaptation have been used to transfer Wi-Fi sensing models with limited target-domain labels, while RFBoost improves robustness through physically grounded augmentation [25,26]. Federated multi-source adaptation extends this concern to distributed radio sensing, and practical Wi-Fi sensing platforms demonstrate that sensing models must operate across changing hardware and channel conditions [27,28,29]. This robustness literature has two implications for privacy-preserving sensing. First, a private semantic that becomes difficult to infer under one clean evaluator may remain recoverable by a model trained for noisy, incomplete, or shifted CSI. Second, a protected representation may remain useful to an authorized service after the downstream model is adapted to that representation. Privacy evaluation should therefore distinguish failure of a fixed evaluator from removal of the underlying semantics and should examine both adaptive private-semantic inference and authorized protected-domain learning.

### 2.2. Privacy Threats and Intervention Layers

As wireless sensing becomes more capable, channel measurements, beamforming feedback, and integrated sensing and communication (ISAC) signals create additional privacy and security surfaces. Recent studies have examined secure ISAC, generative safeguarding for wireless sensing, practical adversarial manipulation of Wi-Fi sensing, identity inference from beamforming feedback, and person recognition from propagation signatures [30,31,32,33,34]. Although these attacks differ in signal source and adversarial objective, they support a common conclusion: wireless information collected for communication or authorized sensing may enable secondary inference beyond the intended task.

Existing defenses can be distinguished by where they intervene. Physical- and channel-layer approaches modify the signal before or during channel estimation. The OpenWiFi CSI fuzzer imposes an artificial channel response that is recoverable by an authorized receiver, while later CSI-fuzzing and dynamic-obfuscation systems modify training fields, virtual channel responses, or complete wireless frames [10,11,12]. MIMOCrypt protects multi-user Wi-Fi sensing through multi-antenna encryption, and ScatterShield uses backscatter tags to disrupt unauthorized sensing [13,14]. These methods can protect the sensing channel before raw CSI reaches an unauthorized observer, but they generally require control over transmitters, firmware, antennas, packet pilots, channel filters, external devices, or authorized recovery procedures.

Data- and model-level defenses instead modify calibration samples or learning behavior. Poison2Cure manipulates calibration data to suppress private sensing capability while retaining useful recognition [15]. This strategy avoids changes to communication hardware, but protection depends on access to the calibration or training process and is tied to predefined private activity classes. It does not generate a protected view of each acquired CSI sample for downstream release.

Representation-level protection occupies a different deployment point. It is applicable after CSI has been acquired by a trusted receiver but before the representation is transferred to storage, analytics, or external services. This setting is particularly relevant when the sensing operator controls the acquisition side but cannot grant every downstream component access to all semantics contained in raw CSI. The central question is therefore not how to conceal the entire wireless channel, but how to regulate semantic accessibility in the representation that crosses the trust boundary. This distinction also prevents an unfair comparison across layers: physical-layer defenses are appropriate when the transmission and authorized recovery paths can be controlled, whereas representation-level defenses address post-acquisition release from a trusted system. These cross-layer differences and their deployment implications are summarized in Table 1.

### 2.3. Representation-Level Semantic Protection

Early representation-level studies formulated CSI privacy as adversarial signal generation. A transformed CSI representation was optimized to mislead a protected semantic classifier while retaining another recognition task [8,9]. This line of work made two important contributions. It recognized that CSI is multi-semantic, and it showed that privacy protection need not make every downstream task fail. These ideas provide the closest conceptual foundation for the present study.

However, existing adversarial CSI transformations do not fully address policy-conditioned selective release. First, the protected semantic is generally fixed during training. Supporting identity-only, location-only, and joint protection therefore requires separate transformations or retraining rather than an explicit runtime policy. Second, the generated modification is applied to the representation as a whole. Even when the transformation is learned, the model does not explicitly separate the allocation of intervention over the time–subcarrier plane from the signed modification applied at those positions. Third, the relationship between retained raw CSI and the released protected representation is usually not formalized as a trusted-boundary data-flow protocol.

A further limitation concerns evaluation scope. Existing adversarial CSI studies commonly assess the generated representation using the classifier or evaluator that defines the protection objective, or a closely related surrogate. Such evidence establishes evaluator-specific suppression, but it does not determine whether private semantics remain learnable by a new classifier trained on labeled protected-domain data. Resistance to post-protection retraining is therefore a distinct empirical question rather than an automatic consequence of low accuracy for the original evaluator. This distinction motivates the architecture-diverse, same- and cross-protector attacker evaluation adopted in this study.

PCGM-Net addresses this narrower representation-release gap. An explicit two-dimensional policy conditions one model to protect identity, location, or both. A learned soft gate determines where intervention is applied, while a bounded residual patch determines how the selected positions are transformed. A keep objective discourages unnecessary suppression of a private semantic not selected by the active policy. Raw CSI is retained inside the trusted acquisition or edge-processing boundary, and only the generated view is released. The framework is therefore designed for policy-controlled inference-time shielding rather than cryptographic secrecy or information-theoretic anonymization.

### 2.4. Generative Wireless Modeling and Comparative Positioning

Generative learning has been introduced into wireless signal synthesis, data augmentation, sensing, and security. RF-Diffusion demonstrates that diffusion models can learn structured radio distributions in time–frequency space, while diffusion-based sensing and generative augmentation use learned signal distributions to improve recognition or data availability [35,36,37]. Generative models have also been investigated for safeguarding wireless sensing and ISAC systems [31]. These studies establish that neural generators can produce structured radio signals rather than unstructured noise.

Generation alone, however, does not provide selective semantic release. Wireless generative models commonly synthesize complete signals, augment training data, or construct safeguarding waveforms. PCGM-Net instead transforms CSI that has already been acquired inside a trusted system. The generator is conditioned on a semantic policy and decomposed into two outputs: a position-wise soft mask and a bounded residual patch. The former provides relative localization with respect to an ungated full-map transformation; the latter supplies the modification content.

Table 1 compares representative defenses across intervention layer, protected object, runtime semantic control, source retention, and deployment dependency. Three distinctions are important. First, physical- and channel-layer defenses intervene before or during acquisition and generally require control over the transmitter, firmware, antennas, packet fields, external devices, or an authorized recovery path; PCGM-Net instead operates after CSI has been acquired inside a trusted system. Second, existing data-, model-, and representation-level methods generally protect predefined targets and do not jointly provide runtime policy switching and a learned local gate over the acquired CSI map. Third, PCGM-Net explicitly separates retained raw CSI from the policy-conditioned representation released downstream. These differences define a complementary post-acquisition release setting rather than a claim of universal superiority over methods deployed at other layers.

## 3. Problem Formulation

### 3.1. Multi-Semantic CSI Representation

For an orthogonal frequency-division multiplexing (OFDM) Wi-Fi link, the received signal on subcarrier *k* at time *t* is

(1) $$Y_{k}\left(t\right)=H_{k}\left(t\right)X_{k}\left(t\right)+N_{k}\left(t\right),$$

where $X_{k}\left(t\right)$ and $Y_{k}\left(t\right)$ are the transmitted and received signals, $N_{k}\left(t\right)$ is additive noise, and $H_{k}\left(t\right)\in C$ is the complex channel response. In a multipath environment,

(2) $$H_{k}\left(t\right)=\sum_{l=1}^{L}\alpha_{l}\left(t\right)e^{-j2\pi f_{k}\tau_{l}\left(t\right)},$$

where $\alpha_{l}\left(t\right)$ and $\tau_{l}\left(t\right)$ denote the complex attenuation and propagation delay of path *l*, respectively. Human motion changes the dynamic propagation paths, whereas transmitter–receiver geometry and the surrounding environment shape the spatial multipath structure. CSI can therefore encode activity-, identity-, and location-related information simultaneously.

Let $P_{\mathrm{pre}}$ denote trusted preprocessing and let $N_{\mathrm{tr}}$ denote normalization whose statistics are estimated exclusively from the training subset. The real-valued source representation used by the learning system is

(3) $$x=N_{\mathrm{tr}}\;\left(P_{\mathrm{pre}}\;\left({\left[H_{k}\left(t\right)\right]}_{\begin{array}{c} t=1,\ldots ,T\\ k=1,\ldots ,K \end{array}}\right)\right)\in R^{T\times K},$$

where *T* and *K* denote the temporal and subcarrier dimensions. Throughout Section 3 and Section 4, *x* denotes this unmodified normalized source map. The source map is retained inside the trusted sensing boundary; the method does not require it to be overwritten or reconstructed.

Let $y_{\mathrm{act}}$, $y_{\mathrm{id}}$, and $y_{\mathrm{loc}}$ denote the activity, identity, and location labels. For each task s∈S={act,id,loc}, a semantic evaluator produces logits

(4) $$F_{s}\left(q\right)\in R^{n_{s}},$$

for an evaluator input *q*, where $n_{s}$ is the number of classes for task *s*. Activity is the authorized semantic in the considered safety-monitoring service. Identity and location are private whenever the downstream recipient is not authorized to receive them.

### 3.2. Policy-Conditioned Selective-Release Problem

The privacy policy is

(5) $$p=\left[p_{\mathrm{id}},p_{\mathrm{loc}}\right],\;p_{\mathrm{id}},p_{\mathrm{loc}}\in \left\{0,1\right\},$$

where $p_{s}=1$ means that private semantic *s* is selected for suppression and $p_{s}=0$ means that it should remain available. The supported policy set is

(6) Π={[1,0],[0,1],[1,1]},

corresponding to identity-only, location-only, and joint protection. The unprotected case is represented directly by the source map *x*; the untrained policy [0,0] is not invoked to obtain an identity transformation.

Operationally, identity-only protection can support a service that requires room or work-zone context for incident management but is not authorized to identify the worker. Location-only protection can support a service that is permitted to verify an authorized worker but should not receive sensitive room or work-zone information. Joint protection applies when the downstream service requires only the activity event. These examples describe configurable release permissions rather than universal privacy rules; the deployed policy should be determined by the service purpose, data-minimization requirements, and the organization’s authorization structure.

Given *x* and an operational policy p∈Π, the trusted side generates

(7) $$x^{\prime }=T_{\theta }\left(x,p\right)$$

and releases only $x^{\prime }$ to downstream services. The source map *x* remains available inside the trusted system.

The selective-release objective has four components. It should preserve authorized activity utility, suppress each private semantic selected by *p*, preserve each unselected private semantic to enforce policy selectivity, and constrain released-view modification. At a generic level,

(8) $$\min_{\theta }\;E_{\left(x,y\right)∼D_{\mathrm{tr}},\;p∼\mathrm{Unif}\left(\Pi \right)}\left[R_{\mathrm{act}}+\sum_{s\in \{\mathrm{id},\mathrm{loc}\}}\left(p_{s}R_{\mathrm{priv}}^{s}+\left(1-p_{s}\right)R_{\mathrm{keep}}^{s}\right)+R_{\mathrm{reg}}\right],$$

where $y=(y_{\mathrm{act}},y_{\mathrm{id}},y_{\mathrm{loc}})$, $D_{\mathrm{tr}}$ denotes the training distribution, and one of the three policies in Π is sampled independently and uniformly for each training sample. Section 4.4 specifies the evaluator-margin, cross-entropy, soft-mask, and distortion terms used to instantiate these quantities.

Source retention and released-view similarity are distinct. Source retention means that the unmodified source representation is preserved inside the trusted boundary. Regularization constrains the transformation applied to the current generator input but does not establish numerical equivalence, propagation-level validity, or physical equivalence.

### 3.3. Threat Model and Trust Assumptions

In the primary threat model, the adversary is instantiated by a validation-selected, degradation-aware multi-task evaluator applied to the released representation $x^{\prime }$. The evaluator cannot access the source map *x*, the trusted source-CSI store, or the protected execution environment. The same evaluator supplies the semantic objectives during generator optimization and is used for the primary fixed-evaluator analysis. It is trained on unprotected CSI under clean, mild, and moderate degradation conditions, selected using validation data only, frozen before PCGM-Net training, and never updated using protected CSI.

A stronger post-protection adversary obtains labeled protected-domain training data and trains a new private-semantic classifier from scratch. The strongest evaluated setting uses outputs generated by the same deployed protector; transfer to independently trained protectors is additionally examined. PCGM-Net does not claim cryptographic secrecy, differential privacy, irreversible information removal, resistance to complete labeled same-protector retraining, or protection after compromise of the trusted boundary. It is evaluated as a policy-selective inference-time release mechanism under a bounded and evaluator-dependent threat model.

## 4. Proposed Method

### 4.1. Local Mask–Patch Transformation

Equation (7) is implemented by the local mask–patch transformation below. Let $v\in R^{T\times K}$ denote the current input to the generator. For clean operation, v=x; during degradation-aware training, *v* is an augmented observation defined in Section 4.5. Given *v* and policy *p*, PCGM-Net predicts a position-wise soft mask

(9) $$M_{\theta }\left(v,p\right)\in {\left[0,1\right]}^{T\times K}$$

and a bounded residual patch

(10) $$\Delta_{\theta }\left(v,p\right)\in {\left[-\epsilon ,\epsilon \right]}^{T\times K},\;\epsilon >0.$$

The released representation is

(11) $$x^{\prime }=v+M_{\theta }\left(v,p\right)⊙\Delta_{\theta }\left(v,p\right).$$

The mask is a continuous intervention-intensity map rather than a binary coverage indicator. It determines where the residual is weighted, whereas the patch determines the signed modification. Locality is therefore defined relative to an ungated full-map residual. Equation (11) uses the current generator input as the residual backbone but does not imply numerical or physical equivalence between *v* and the released representation $x^{\prime }$.

### 4.2. Policy-Conditioned Architecture

The two policy components are expanded into constant spatial maps,

(12) $$P_{\mathrm{id}}=p_{\mathrm{id}}1_{T\times K},\;P_{\mathrm{loc}}=p_{\mathrm{loc}}1_{T\times K},$$

and concatenated with the single-channel generator input:

(13) $$u\left(v,p\right)=\mathrm{Concat}\;\left(v,P_{\mathrm{id}},P_{\mathrm{loc}}\right)\in R^{3\times T\times K}.$$

The implemented shared encoder contains four 3×3 convolutional blocks with channel widths 32, 32, 64, and 64, each followed by batch normalization and rectified linear unit (ReLU) activation. It preserves the T×K spatial resolution and produces

(14) $$f_{\theta }=E_{\theta }\;\left(u(v,p)\right).$$

The mask and patch heads each use a 3×3 convolution from 64 to 32 channels, a ReLU activation, and a 1×1 output convolution. Their outputs are

(15) $$M_{\theta }\left(v,p\right)=\sigma \;\left(D_{\theta }^{M}\left(f_{\theta }\right)\right),\;\Delta_{\theta }\left(v,p\right)=\epsilon \tanh\;\left(D_{\theta }^{\Delta }\left(f_{\theta }\right)\right).$$

This input-level conditioning and shared-encoder, dual-head design separates intervention allocation from transformation content. It does not assume that the learned mask identifies a unique physical origin of activity-, identity-, or location-related evidence.

### 4.3. Shared Multi-Task Evaluator

The validation-selected semantic evaluator uses a shared convolutional backbone and three task-specific heads. For a generic evaluator input *q*,

(16) $$h=E_{\phi_{E}}\left(q\right),\;F_{s}\left(q\right)=C_{s}\left(h;\phi_{s}\right),\;s\in S,$$

where $F_{s}\left(q\right)$ denotes the logits for task *s*. The implemented backbone uses convolutional channel widths 32, 64, 128, and 128; the first two blocks include 2×2 max pooling, and the last block is followed by adaptive average pooling. A 128-dimensional projection with dropout 0.25 feeds linear activity, identity, and location heads with 7, 10, and 6 outputs, respectively.

**Figure 2:**
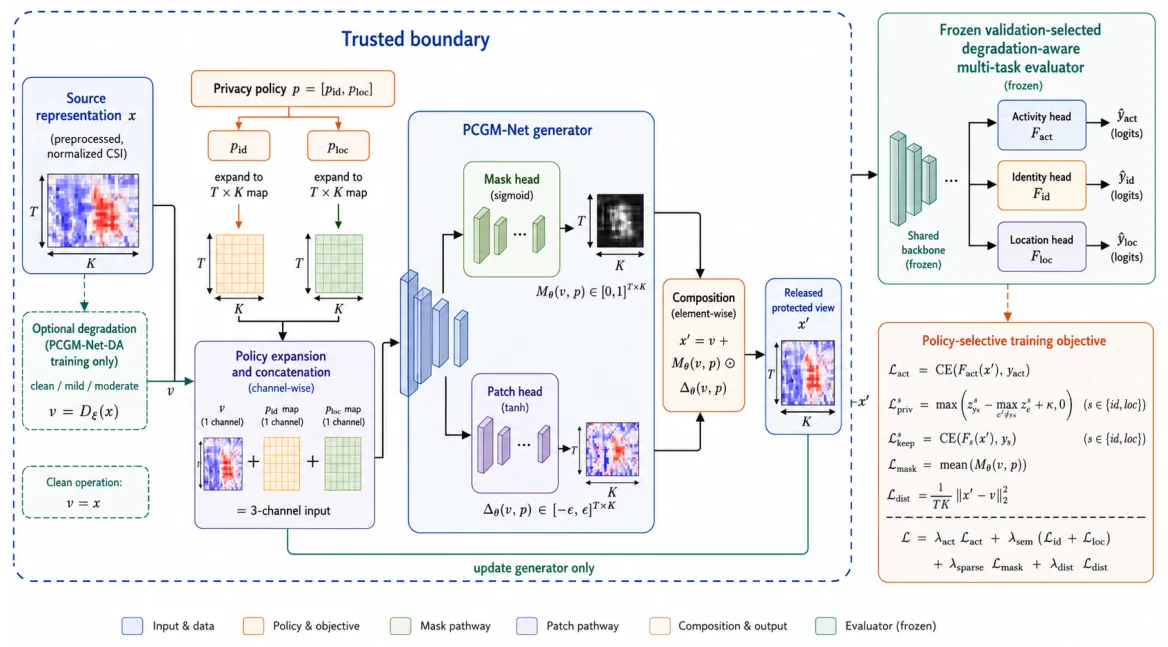
Implemented PCGM-Net architecture. Inside the trusted boundary, the identity and location policy components are expanded into constant spatial maps and concatenated with the current generator input *v*. A shared encoder feeds a sigmoid mask head and a tanh patch head, producing $M_{\theta }\left(v,p\right)$ and the bounded residual $\Delta_{\theta }\left(v,p\right)$. Their gated residual composition yields $x^{\prime }=v+M_{\theta }\left(v,p\right)⊙\Delta_{\theta }\left(v,p\right)$, and only $x^{\prime }$ is released. The validation-selected multi-task evaluator remains frozen during generator optimization; post-protection attackers are evaluated separately. Arrows indicate data flow or optimization dependencies, colors correspond to the pathway categories shown in the legend, and ellipses indicate omitted intermediate layers.

During evaluator training, a degradation condition is sampled uniformly per mini-batch from the clean, mild, and moderate conditions. Let $q=D_{\xi }\left(x\right)$ denote the resulting unprotected evaluator input. The evaluator is optimized by

(17) $$L_{\mathrm{eval}}=\sum_{s\in S}\alpha_{s}\;\mathrm{CE}\;\left(F_{s}\left(q\right),y_{s}\right),$$

where CE denotes cross-entropy, $\alpha_{s}\geq 0$, and the implementation sets $\alpha_{s}=1$ for all s∈S. After validation-only checkpoint selection, all evaluator parameters $\phi =\{\phi_{E},\phi_{\mathrm{act}},\phi_{\mathrm{id}},\phi_{\mathrm{loc}}\}$ are frozen, and generator training updates only θ. Figure 2 summarizes the implemented data flow, including the shared encoder, dual output heads, gated composition, trusted execution boundary, and frozen multi-task evaluator.

### 4.4. Policy-Selective Objective

Authorized activity is preserved by

(18) $$L_{\mathrm{act}}=\mathrm{CE}\;\left(F_{\mathrm{act}}\left(x^{\prime }\right),y_{\mathrm{act}}\right).$$

For private semantic s∈{id,loc}, let $z^{s}=F_{s}\left(x^{\prime }\right)$ denote its logits. The evaluator-dependent untargeted margin loss is

(19) $$L_{\mathrm{priv}}^{s}=\max\;\left(z_{y_{s}}^{s}-\max_{c\neq y_{s}}z_{c}^{s}+\kappa ,0\right),\;\kappa \geq 0,$$

where minimizing the loss encourages at least one incorrect-class logit to exceed the true-class logit by the margin κ. The retention loss for an unselected private semantic is

(20) $$L_{\mathrm{keep}}^{s}=\mathrm{CE}\;\left(F_{s}\left(x^{\prime }\right),y_{s}\right).$$

The policy-selective semantic term is

(21) $$L_{s}=p_{s}w_{\mathrm{priv}}^{s}L_{\mathrm{priv}}^{s}+\left(1-p_{s}\right)w_{\mathrm{keep}}^{s}L_{\mathrm{keep}}^{s},$$

where $w_{\mathrm{priv}}^{s}\geq 0$ and $w_{\mathrm{keep}}^{s}\geq 0$ control task-specific suppression and retention.

The soft-mask mean and released-view distortion penalties are

(22) $$L_{\mathrm{mask}}=\frac{1}{TK}\sum_{i=1}^{T}\sum_{j=1}^{K}M_{\theta }{\left(v,p\right)}_{i,j},\;L_{\mathrm{dist}}=\frac{1}{TK}{∥x^{\prime }-v∥}_{2}^{2}.$$

The first term penalizes average intervention intensity. It encourages lower average mask activation but does not guarantee binary values, exact sparsity, or a uniquely localized semantic region. The complete generator objective is

(23) $$L=\lambda_{\mathrm{act}}L_{\mathrm{act}}+\lambda_{\mathrm{sem}}\left(L_{\mathrm{id}}+L_{\mathrm{loc}}\right)+\lambda_{\mathrm{sparse}}L_{\mathrm{mask}}+\lambda_{\mathrm{dist}}L_{\mathrm{dist}},$$

where all λ coefficients are nonnegative. The semantic losses quantify behavior with respect to the frozen evaluator. The soft-mask and distortion terms regulate optimization behavior but do not guarantee physical plausibility or monotonic changes in post hoc structural statistics.

### 4.5. Degradation-Aware Training and Trusted Release

Let $D_{\xi }$ denote the stochastic degradation operator and let

(24) $$v=D_{\xi }\left(x\right),\;x^{\prime }=v+M_{\theta }\left(v,p\right)⊙\Delta_{\theta }\left(v,p\right).$$

For degradation-aware PCGM-Net (PCGM-Net-DA) training, one condition is sampled uniformly per mini-batch from clean, mild, and moderate degradation; stochastic gain, noise, point-dropout, time-dropout, and subcarrier-dropout realizations are then generated within the selected condition. Severe degradation is excluded from fitting and checkpoint selection and is reserved as an unseen stress condition. The clean condition corresponds to the identity operator, $D_{\xi }\left(x\right)=x$.

At inference, the synthetic degradation operator is not required. PCGM-Net receives the currently observed, preprocessed, and normalized CSI representation and executes within a trusted receiver host, protected access-point process, or colocated edge service. The unmodified source representation *x* remains inside the trusted system, while downstream components receive only $x^{\prime }$. The mechanism complements rather than replaces secure execution, authentication, access control, transport protection, and encryption at rest.

## 5. Experimental Evaluation and Discussion

This section presents the experimental protocol, results, and interpretation. The evaluation examines seven questions: whether unprotected CSI exposes identity and location; whether one policy-conditioned model can selectively regulate their release; how the privacy–utility operating point changes under non-ideal CSI and for safety-critical activities; whether the mask, residual patch, policy input, and objective terms have distinct functions; how the reference configuration compares with alternative hyperparameters and learned full-map sanitizers; how protection changes the released representation and its compatibility with independently trained downstream models; and whether the measured overhead permits low-latency execution on the tested platforms.

### 5.1. Experimental Protocol

Experiments used a processed multi-semantic Wi-Fi channel state information (CSI) dataset with synchronized activity, identity, and location labels. Ten participants performed seven activities—falling, standing, sitting, lying, jumping, phone use, and leaving the monitored area—in six laboratory-scale indoor rooms. Identity labels are anonymized participant indices, whereas location labels correspond to the six rooms.

**Figure 3:**
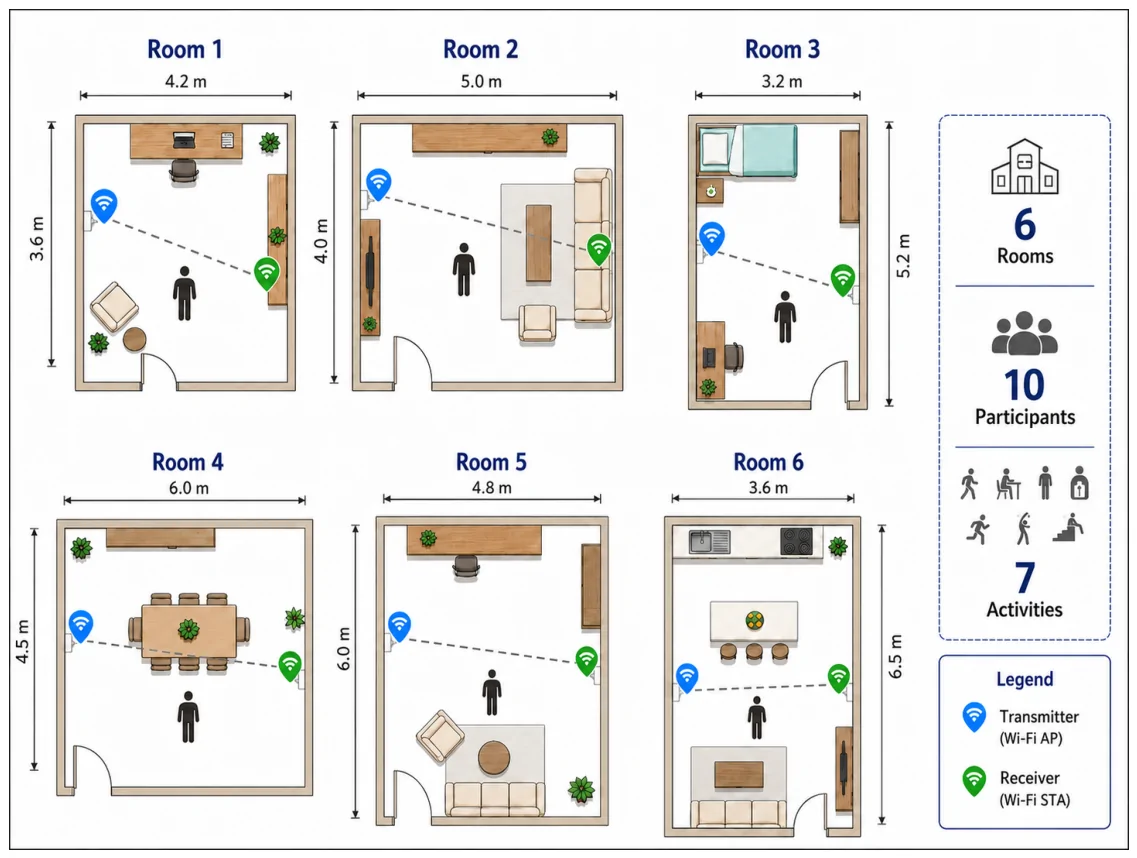
Laboratory-scale CSI collection layout. Six rooms define the location classes, and ten anonymized participants perform seven activities within device-free transmitter–receiver links. AP denotes access point, and STA denotes station.

The original dataset contains 30,660 training samples and 4200 test samples. Each sample is stored as a 90×20 subcarrier-by-time array, transposed to a 20×90 time-by-subcarrier map, and reshaped as a single-channel tensor. The original training partition comprises 420 joint activity–identity–location strata, each containing 73 samples. A fixed stratified split assigned 62 samples per stratum to training and 11 to validation, yielding 26,040 training samples and 4620 validation samples. Normalization statistics were estimated exclusively from the training subset. Figure 3 illustrates the laboratory-scale CSI collection layout used to define the six location classes.

The original test partition was excluded from model fitting, coefficient selection, epoch selection, and checkpoint selection and was accessed only after all checkpoints participating in a given comparison had been fixed. This test-set-isolated design prevents selection leakage but guarantees sample-level separation only. All activities, participants, and rooms occur in both original partitions, and reliable acquisition-sequence, trajectory, session, and device identifiers are unavailable. The results therefore characterize closed-set, in-distribution semantic leakage and protection rather than cross-subject, cross-room, cross-session, or cross-device generalization.

Activity recognition was treated as the authorized utility task; identity and location recognition were treated as privacy-sensitive inference tasks. Hereafter, “raw CSI” in result tables denotes the unprotected preprocessed source representation rather than the original complex channel measurements. Accuracy and the macro-averaged F1 score (macro-F1) were reported for all three tasks. Recall and false-negative rate (FNR) were additionally reported for falling and leaving the monitored area. Representation modification was summarized by mean mask intensity and normalized mean squared error (NMSE) after inverse normalization. Mask intensity is the continuous mean of the soft gate rather than a binary coverage ratio. Structural diagnostics included perturbation-to-signal power, correlations between the raw and protected aggregate temporal and subcarrier profiles, temporal-spectrum cosine similarity and temporal-spectrum Jensen–Shannon (JS) divergence, maximum absolute residual, and lag-1 temporal and subcarrier differences. These diagnostics characterize representation-level change but do not establish propagation-level validity or physical equivalence; complete structural and mask diagnostics are reported in Supporting Information Tables S7 and S8.

The semantic evaluator uses a shared convolutional backbone with separate activity, identity, and location heads. PCGM-Net-DA was optimized for 40 epochs using AdamW, a learning rate of $10^{-3}$, weight decay of $10^{-4}$, batch size 256, and cosine annealing. The reference coefficients were selected through pilot runs using the training and validation subsets only; the test partition was not used for coefficient selection. Selection aimed to keep the loss terms on comparable numerical scales, preserve authorized activity utility, and avoid trivial solutions in which the private semantics remained unchanged or the released representation was excessively distorted. The resulting coefficients define a validation-chosen reference operating point rather than a statistically or globally optimal configuration. The reference setting used ϵ=3.0, $\lambda_{\mathrm{act}}=3.0$, $\lambda_{\mathrm{sem}}=1.0$, $\lambda_{\mathrm{sparse}}=0.015$, and $\lambda_{\mathrm{dist}}=0.02$. Identity and location privacy weights were 2.0 and 8.0, respectively, and both keep weights were 1.0. Complete coefficient definitions and degradation settings are provided in Supporting Information Tables S1 and S2.

Checkpoint selection maximized a validation-only score averaged over clean and moderate conditions. Activity retention, preservation of an unselected private semantic, and suppression of each selected semantic received equal score-level importance. Severe degradation was excluded from model fitting and selection. The selected full-model checkpoints occurred at epochs 40, 40, and 37 for model seeds 42, 123, and 2026, respectively.

The full model, component variants, learned sanitization baselines, and sensitivity configurations used model-training seeds 42, 123, and 2026. Clean results are reported as mean ± sample standard deviation across the three independently trained models. For degraded protected-CSI results, each model was first averaged over degradation seeds 42, 43, and 44; mean and sample standard deviation were then calculated across model-training seeds. For the fixed evaluator applied to unprotected CSI, degraded-result variability reflects degradation realizations only. Post-protection attacker results use three attacker-training seeds, and independent downstream-activity results use three activity-model seeds. Sample-level hypothesis tests were not performed because CSI samples sharing participants, rooms, and acquisition processes are not independent experimental replicates.

Two representative learned full-map protect-both baselines, eight component variants, and a one-factor-at-a-time sensitivity analysis used the same split, normalization, epoch budget, validation-only selection, and model seeds as the reference model. The gradient reversal layer (GRL) autoencoder combined activity preservation with gradient-reversal identity and location heads, whereas the entropy-regularized autoencoder preserved activity while increasing the predictive entropy of the private-task heads. These baselines represent the corresponding learning mechanisms rather than exact reproductions of a particular published implementation. The adaptive-attacker protocol trained 12 single-task classifiers from scratch on labeled outputs generated by protector seed 42, using two architectures—a residual convolutional neural network (CNN) and a temporal bidirectional gated recurrent unit (BiGRU)—two private tasks, and three attacker seeds. These models were tested on the same protector and on independently trained protectors 123 and 2026. An independent BiGRU activity model was trained on raw data, protected data, or mixed-domain data. For mixed-domain training, each training sample independently used the protected representation with probability 0.5 and the corresponding raw representation otherwise. The implementation used Python 3.11 and PyTorch 2.10. Profiling was conducted on a Lenovo Legion R9000P ADR10H notebook (Lenovo Group Limited, Beijing, China) equipped with an AMD Ryzen 9 8945HX processor (Advanced Micro Devices, Inc., Santa Clara, CA, USA) (32 logical cores), 32 GB of system memory, and an NVIDIA GeForce RTX 5070 Ti Laptop graphics processing unit (GPU) (NVIDIA Corporation, Santa Clara, CA, USA) with 12 GB of dedicated video memory. GPU profiling used the discrete NVIDIA GPU, whereas central processing unit (CPU) profiling used the same evaluator and protector on the Ryzen 9 8945HX processor. Energy consumption and thermal behavior were not measured. Supporting Information Tables S1–S14 provide the complete coefficient and degradation settings, additional moderate-degradation results for the principal architecture and degradation-training variants, complete policy-selectivity diagnostics and clean- and moderate-condition sensitivity results, structural and mask diagnostics, adaptive-attacker results, downstream-activity results, and CPU-profiling results.

ChatGPT (OpenAI OpCo, LLC, San Francisco, CA, USA; https://chatgpt.com/; accessed on 8 August 2026) was used only after the experiments for language editing, structural refinement, and formatting assistance; it was not used to generate data, execute analyses, select models, or determine scientific conclusions.

### 5.2. Selective Semantic Release

The evaluator recovered substantial private information from unprotected CSI (Table 2). Under clean input, identity and location accuracies were 97.95% and 100.00%, respectively. Under severe degradation, activity macro-F1 fell to 45.62%, whereas location macro-F1 remained 97.44%. Non-ideal signal conditions therefore did not reliably eliminate private-semantic recoverability.

The three requested policies produced distinct outcomes under clean CSI (Table 3). Identity-only protection reduced identity accuracy to 8.92% while retaining 99.50% location accuracy. Location-only protection reduced location accuracy to 0.38% while retaining 80.19% identity accuracy. Joint protection reduced identity and location accuracies to 6.09% and 0.29%, respectively, while retaining 74.79% activity accuracy. Macro-F1 values showed the same policy-dependent pattern.

These results establish the first requirement of the proposed framework: a single model changes its released semantics according to the requested policy rather than applying one fixed privacy transformation. The claim remains evaluator-dependent; the low private-task accuracies quantify reduced recoverability for the validation-selected evaluator and do not imply irreversible information removal.

### 5.3. Robustness and Safety-Critical Utility

Under the protect-both policy, activity accuracy decreased from 74.79% under clean CSI to 57.49% under mild degradation, 42.51% under moderate degradation, and 27.91% under severe degradation (Table 4). Identity accuracy increased to 13.01%, 17.46%, and 16.94%, respectively; location accuracy increased to 1.35%, 3.12%, and 8.74%. Mask intensity remained near 51%, whereas NMSE ranged from 0.350 to 0.402. Because degradation changes both the input reference and its signal energy, cross-condition NMSE is descriptive rather than a monotonic measure of protection strength.

Class-specific errors exposed limitations that average seven-class accuracy obscures (Table 5). Under clean joint protection, FNR was 19.22% for falling and 21.44% for leaving the monitored area. Under moderate degradation, these values increased to 51.22% and 47.00%; under severe degradation, they reached 67.98% and 54.28%.

Degradation-aware training improved the non-ideal-CSI operating point, but it did not make the framework uniformly robust. Moderate and severe degradation produced safety-critical error rates that are unsuitable for autonomous high-consequence decisions. Practical use therefore requires channel-quality monitoring, abstention or rejection logic, and a trusted fallback path. Severe degradation should be treated as an out-of-operating-region stress condition rather than as evidence of deployment robustness.

### 5.4. Mechanistic Validation of Local and Policy-Conditioned Transformation

Removing the privacy-margin objective restored identity and location accuracies to 98.55% and 100.00%, respectively, while reducing NMSE to 0.0016 (Table 6). The residual-patch-only variant retained 77.40% activity accuracy and low evaluator accuracy for identity and location, but applied its residual over the complete map and produced higher NMSE. The mask-only variant yielded lower NMSE but retained only 56.84% activity accuracy.

The branch ablations clarify the primary architectural contribution. The residual branch supplies most of the semantic transformation capacity, whereas the mask controls where that residual is applied relative to an ungated full-map transformation. Because the mean mask intensity remains near 50%, the evidence supports relative localization rather than extreme sparsity, binary semantic segmentation, or identification of a unique physical privacy region.

Policy conditioning and the keep loss governed selective control rather than suppression alone (Table 7). Removing the policy input produced identical output statistics for identity-only, location-only, and joint requests. Removing the keep loss caused location-only protection to reduce the unselected identity accuracy from 80.19% to 12.10%.

These results distinguish three mechanisms that should not be conflated: the privacy-margin objective induces evaluator-specific suppression; the policy input determines which semantics are selected; and the keep term limits unnecessary suppression of semantics that the policy permits. The complete model is therefore supported by mechanism-specific evidence rather than by aggregate privacy accuracy alone.

### 5.5. Hyperparameter Sensitivity and Baseline Positioning

Privacy weighting produced the clearest directional privacy–utility trade-off (Table 8). Under clean CSI, reducing the privacy scale to 0.5 increased activity accuracy to 83.24% but increased identity accuracy to 10.97%. Increasing the scale to 1.5 reduced activity accuracy to 70.06% while reducing identity and location accuracies to 4.44% and 0.13%. The residual bound produced an amplitude–support compensation: reducing ϵ from 3.0 to 1.5 increased clean-condition mask intensity from 51.19% to 67.09%, whereas increasing ϵ to 4.5 reduced it to 37.03% (Supporting Information Table S5). Isolated changes in sparsity and distortion weights produced smaller and non-monotonic differences.

The reference setting is therefore best interpreted as a validation-selected intermediate operating point, not as a uniquely or globally optimal parameter combination. Privacy weighting acts as the most interpretable control of the privacy–utility frontier, whereas the sparsity and distortion terms interact with the other objectives and do not provide one-to-one control over the corresponding test statistics.

The learned full-map baselines occupied different privacy–utility operating points (Table 9). The GRL autoencoder’s low private-task accuracies were accompanied by clean activity accuracy of 13.90%, close to the seven-class chance level of 14.29%, and NMSE above 4.3. The entropy-regularized autoencoder retained higher activity accuracy and lower NMSE than PCGM-Net-DA but permitted greater private-semantic recoverability, particularly for location. Under moderate degradation, it retained 66.01% activity accuracy but yielded 23.39% location accuracy, compared with 42.51% and 3.12% for PCGM-Net-DA.

The entropy-regularized autoencoder and PCGM-Net-DA represent distinct non-dominated privacy–utility operating points. The entropy baseline retains higher activity utility and lower NMSE but permits substantially greater private-semantic recoverability, particularly for location. The GRL autoencoder does not provide a practically useful operating point in this evaluation because its low private-task accuracies are accompanied by near-chance activity recognition and severe representation distortion. PCGM-Net-DA additionally provides runtime policy selection and learned local gating, but it does not maximize activity performance.

### 5.6. Structural Characteristics of the Protected Representation

Structural diagnostics revealed anisotropic preservation rather than physical equivalence (Table 10). Under clean joint protection, subcarrier-profile correlation was 0.788 and temporal-spectrum cosine similarity was 0.724, whereas temporal-profile correlation was only 0.039. Under moderate degradation, subcarrier correlation remained 0.787 and temporal-spectrum cosine similarity increased to 0.861, while temporal-profile correlation remained low at 0.113. The perturbation-to-signal power ratio was −4.57 dB under clean CSI and −4.89 dB under moderate degradation.

The protected tensor therefore retains part of the aggregate subcarrier and temporal-spectrum structure while substantially reorganizing the temporal profile. Higher spectral similarity under moderate degradation reflects comparison with an already degraded reference and should not be interpreted as improved propagation fidelity. The released tensor is best viewed as a learned machine-sensing representation, not as a physically calibrated substitute for raw CSI.

### 5.7. Adaptive Privacy Boundary and Authorized-Utility Adaptation

Labeled same-protector retraining recovered most private semantics (Table 11). Under clean CSI, the residual CNN and temporal BiGRU recovered identity with 99.87% and 98.79% accuracy, respectively, and both recovered location with 100.00% accuracy. These values are far above the corresponding identity and location chance levels of 10.00% and 16.67%. Under severe degradation, same-protector identity accuracy remained 64.09–70.48%, and location accuracy remained 98.28–99.22% (Supporting Information Tables S9 and S10).

Cross-protector transfer was lower but remained architecture dependent and above chance. Under clean CSI, the residual CNN achieved 18.17–20.64% identity accuracy and 41.03–47.58% location accuracy across protectors 123 and 2026. The temporal BiGRU achieved 39.10–39.31% identity accuracy and 74.72–75.09% location accuracy. Uniform logit ensembles further increased temporal BiGRU cross-protector recoverability to approximately 47% for identity and 77% for location (Supporting Information Table S12).

These results define the central privacy boundary. PCGM-Net limits inference by non-adapted evaluators, but it does not erase private information in an information-theoretic or irreversible sense. Labeled protected-domain training can relearn private semantics, and part of this relearned structure transfers across independently trained protectors. Mitigating this stronger threat would require additional measures, such as randomized or rotating transformations, broader adversarial training, restrictions on labeled private data, and conventional cryptographic or access-control mechanisms around the released representation.

The independent activity model showed the complementary utility boundary (Table 12). A model trained only on raw CSI achieved 92.98% accuracy on raw data but 36.04% on same-protector protected data. A protected-domain model achieved 94.09% on same-protector protected data but 42.51% on raw CSI. Mixed-domain training yielded 89.83% accuracy on raw data and 89.76% on protected data, all far above the seven-class chance level of 14.29%.

Authorized activity information therefore remains learnable, but the protected representation constitutes a distinct model domain. A practical downstream service should be calibrated on protected or mixed data generated by the deployed protector rather than assuming that a raw-CSI model will transfer unchanged. Cross-protector performance remained limited: protected-only training yielded approximately 26–27% activity accuracy, whereas mixed-domain training yielded approximately 41% on independently trained protectors (Supporting Information Table S13). Independently trained protectors therefore do not automatically define an interchangeable downstream feature space.

### 5.8. Computational Efficiency and Deployment Implications

The PCGM generator contains 102,946 parameters and requires approximately 368.29 million floating-point operations (FLOPs) per sample under the adopted counting convention. The complete protection-and-recognition pipeline contains 363,385 parameters and requires approximately 434.62 million FLOPs per sample. On the NVIDIA GeForce RTX 5070 Ti Laptop GPU, the batch-size-1 pipeline achieved 2.18 ms mean latency, 2.26 ms 95th-percentile (P95) latency, 12.87 MiB peak memory, and approximately 458 samples per second. Table 13 summarizes these model-complexity and batch-size-1 inference measurements.

At batch size 256, the GPU pipeline required 21.76 ms per batch, corresponding to approximately 11,766 samples per second and 577.89 MiB peak memory. On the AMD Ryzen 9 8945HX CPU, batch-size-1 pipeline latency was 4.25 ms with one thread, 1.97 ms with four threads, and 1.49 ms with eight threads; the eight-thread P95 latency was 1.81 ms. A repeated synthetic GPU optimization-step microbenchmark yielded an extrapolated estimate of approximately 9.50 s per epoch and 6.3 min for 40 epochs; these values are not measured end-to-end wall-clock training times.

These measurements support low-latency execution on the tested notebook-class GPU and CPU backends, but they do not establish energy efficiency or feasibility on a specific industrial access point or embedded gateway. Energy and thermal behavior were not measured, and GPU and CPU latency should not be interpreted as an energy-efficiency comparison. The intended execution point is immediately after CSI extraction and preprocessing within a trusted receiver host, protected access-point process, or colocated edge service. Raw CSI must not be exposed to external storage or analytics before transformation, and PCGM-Net complements rather than replaces secure execution, authentication, access control, and encryption.

## 6. Conclusions

This study introduced PCGM-Net, a policy-conditioned local generative masking framework for selective semantic release in Wi-Fi CSI sensing. To the best of our knowledge, PCGM-Net is the first representation-level Wi-Fi CSI framework to integrate four capabilities in a single design. These capabilities are explicit runtime multi-semantic policies, learned local gating over the time–subcarrier plane, bounded residual modification, and trusted source retention. By separating where intervention is applied from how selected regions are transformed, a single model provides unified and controllable semantic accessibility while retaining the source CSI inside the trusted sensing boundary.

The integrated experimental evidence supports three primary conclusions. First, PCGM-Net provides controllable evaluator-dependent suppression driven by targeted semantic optimization rather than incidental representation corruption, with the architectural and objective components serving distinct and complementary functions. Second, the protected representation preserves learnable authorized activity information, and protected-domain or mixed-domain adaptation can substantially recover downstream utility. Third, the released representation is not irreversibly anonymized: supervised attackers retrained on labeled protected outputs recovered private semantics, positioning PCGM-Net as a policy-controlled inference-time release mechanism for bounded threat settings rather than as cryptographic anonymization.

Practical industrial deployment therefore requires trusted execution before disclosure, restrictions on private-label availability, calibration of authorized downstream models, channel-quality gating for safety-critical decisions, and complementary system security controls. Future work will extend validation to multi-site datasets with session-, device-, and environment-disjoint splits, connect representation-level diagnostics with physical channel fidelity, examine online adaptation and randomized transformations, explore joint hyperparameter optimization, and evaluate energy, thermal, and end-to-end packet-processing performance on embedded edge gateways. 

## Figures and Tables

**Table 1:** Comparison of representative wireless-sensing privacy mechanisms across intervention, semantic control, source retention, and deployment requirements.

Approach	Intervention Layer	Protected Object or Operation	Runtime Semantic Control	Source Retention and External Modification	Main Deployment Dependency
Adversarial CSI transformation [8,9]	Representation	Generated full CSI representation	No runtime policy; no local gate	Raw-source retention not explicit; no external modification	Access to the target evaluator or a surrogate model
OpenWiFi and related CSI fuzzing [10,11]	Physical or channel layer	Artificial channel response or training-field modification	No runtime policy; no local gate	Raw-source retention not applicable; external modification required	Transmitter, firmware, or packet-field control
Dynamic CSI obfuscation [12]	Physical or channel layer	Complete-frame filtering with authorized recovery	No runtime policy; no local gate	Raw-source retention not applicable; external modification required	Channel-filtering and authorized recovery procedure
MIMOCrypt and ScatterShield [13,14]	Physical or external-device layer	Multi-antenna encryption or backscatter disruption	No runtime policy; no local gate	Raw-source retention not applicable; external modification required	Multi-antenna configuration or external tags and devices
Poison2Cure [15]	Data or model layer	Calibration-data intervention for predefined private activities	No runtime policy; no local gate	Raw-source retention not applicable; no external modification	Access to calibration data or the model-training process
Generative secure sensing [31]	Signal or representation layer	Generated safeguarding signal	No runtime policy; no local gate	Raw-source retention not explicit; signal-path integration required	Integration with the sensing or communication signal-generation process
PCGM-Net (this work)	Representation release	Policy-conditioned protected view of acquired CSI	Identity, location, or both; learned local gate	Raw source explicitly retained; no external modification	Trusted receiver-side or edge execution before downstream release

**Table 2:** Semantic performance of the validation-selected evaluator on unprotected CSI. Clean evaluation is deterministic; degraded values are mean ± standard deviation over degradation seeds 42, 43, and 44.

Condition	Act. Acc.	Act. F1	Id. Acc.	Id. F1	Loc. Acc.	Loc. F1
	(%)	(%)	(%)	(%)	(%)	(%)
Clean	93.57	93.58	97.95	97.94	100.00	100.00
Mild	87.23 ± 0.07	87.22 ± 0.07	96.67 ± 0.31	96.67 ± 0.31	100.00 ± 0.00	100.00 ± 0.00
Moderate	74.55 ± 0.61	74.46 ± 0.62	91.44 ± 0.27	91.45 ± 0.27	99.98 ± 0.01	99.98 ± 0.01
Severe	46.02 ± 0.05	45.62 ± 0.10	69.17 ± 1.11	69.14 ± 1.12	97.44 ± 0.43	97.44 ± 0.43

**Table 3:** Policy-selective performance under clean CSI. Values are mean ± sample standard deviation across three independently trained PCGM-Net-DA models.

Policy	Act. Acc.	Act. F1	Id. Acc.	Id. F1	Loc. Acc.	Loc. F1	Mask Intensity	NMSE
	(%)	(%)	(%)	(%)	(%)	(%)	(%)	
Protect identity	83.39 ± 1.64	83.40 ± 1.69	8.92 ± 1.46	7.00 ± 0.82	99.50 ± 0.36	99.50 ± 0.36	42.61 ± 6.46	0.102 ± 0.025
Protect location	77.55 ± 2.91	77.54 ± 2.91	80.19 ± 5.06	79.96 ± 5.07	0.38 ± 0.12	0.64 ± 0.33	49.55 ± 4.39	0.301 ± 0.095
Protect both	74.79 ± 0.96	74.72 ± 1.00	6.09 ± 2.40	5.66 ± 2.05	0.29 ± 0.14	0.43 ± 0.28	51.19 ± 3.70	0.402 ± 0.150

**Table 4:** Joint-protection performance across CSI degradation levels. For degraded conditions, each trained model was first averaged over degradation seeds 42, 43, and 44; values are then mean ± sample standard deviation across the three model-training seeds.

Condition	Act. Acc.	Act. F1	Id. Acc.	Id. F1	Loc. Acc.	Loc. F1	Mask Intensity	NMSE
	(%)	(%)	(%)	(%)	(%)	(%)	(%)	
Clean	74.79 ± 0.96	74.72 ± 1.00	6.09 ± 2.40	5.66 ± 2.05	0.29 ± 0.14	0.43 ± 0.28	51.19 ± 3.70	0.402 ± 0.150
Mild	57.49 ± 0.64	57.37 ± 0.56	13.01 ± 2.96	12.13 ± 2.61	1.35 ± 0.27	2.15 ± 0.79	51.21 ± 3.56	0.384 ± 0.150
Moderate	42.51 ± 1.45	42.19 ± 1.41	17.46 ± 2.10	15.31 ± 1.82	3.12 ± 0.61	4.39 ± 0.74	51.31 ± 3.52	0.368 ± 0.155
Severe	27.91 ± 1.44	26.96 ± 1.61	16.94 ± 1.23	13.64 ± 0.71	8.74 ± 6.65	7.97 ± 4.70	51.61 ± 3.58	0.350 ± 0.165

**Table 5:** Recall and false-negative rate for falling and leaving the monitored area. Raw degraded results summarize degradation-seed variability; protected results summarize model-training variability after averaging degradation realizations within each model.

Condition	Input or Protection Policy	Fall Recall	Fall FNR	Leaving Recall	Leaving FNR
		(%)	(%)	(%)	(%)
Clean	Raw CSI	96.17	3.83	92.67	7.33
Clean	PCGM-Net-DA, protect both	80.78 ± 1.44	19.22 ± 1.44	78.56 ± 3.14	21.44 ± 3.14
Mild	Raw CSI	88.89 ± 0.10	11.11 ± 0.10	89.06 ± 0.79	10.94 ± 0.79
Mild	PCGM-Net-DA, protect both	64.56 ± 4.81	35.44 ± 4.81	64.22 ± 1.38	35.78 ± 1.38
Moderate	Raw CSI	76.61 ± 1.36	23.39 ± 1.36	81.72 ± 0.38	18.28 ± 0.38
Moderate	PCGM-Net-DA, protect both	48.78 ± 7.11	51.22 ± 7.11	53.00 ± 4.30	47.00 ± 4.30
Severe	Raw CSI	47.56 ± 1.17	52.44 ± 1.17	61.78 ± 1.13	38.22 ± 1.13
Severe	PCGM-Net-DA, protect both	32.02 ± 5.18	67.98 ± 5.18	45.72 ± 9.44	54.28 ± 9.44

**Table 6:** Component ablation under clean CSI and the protect-both policy. Values are mean ± sample standard deviation across three independently trained models. For the residual-patch-only variant, 100% mask intensity denotes ungated full-map residual application rather than a learned mask.

Variant	Act. Acc.	Act. F1	Id. Acc.	Loc. Acc.	Mask Intensity	NMSE
	(%)	(%)	(%)	(%)	(%)	
Full PCGM-Net-DA	74.79 ± 0.96	74.72 ± 1.00	6.09 ± 2.40	0.29 ± 0.14	51.19 ± 3.70	0.402 ± 0.150
Mask branch only	56.84 ± 4.75	56.28 ± 5.05	11.52 ± 0.64	0.33 ± 0.08	55.13 ± 1.25	0.235 ± 0.023
Residual-patch branch only	77.40 ± 1.45	77.38 ± 1.41	5.17 ± 1.14	0.33 ± 0.22	100.00 ± 0.00	0.519 ± 0.078
Without privacy-margin loss	93.48 ± 0.17	93.48 ± 0.17	98.55 ± 0.11	100.00 ± 0.00	6.62 ± 0.61	0.0016 ± 0.0003
Without sparsity regularization	76.08 ± 2.18	76.09 ± 2.12	5.97 ± 0.79	0.18 ± 0.11	47.93 ± 3.05	0.418 ± 0.166
Without distortion regularization	74.67 ± 1.54	74.67 ± 1.51	6.68 ± 1.95	0.17 ± 0.05	45.44 ± 5.80	0.370 ± 0.106
Without degradation-aware training	90.51 ± 1.93	90.49 ± 1.91	2.27 ± 1.93	0.33 ± 0.11	47.56 ± 2.19	0.402 ± 0.219

**Table 7:** Compact diagnosis of policy conditioning and unselected-semantic retention under clean CSI. Values are mean ± sample standard deviation across three independently trained models.

Variant	Requested Policy	Act. Acc.	Id. Acc.	Loc. Acc.	Mask Intensity	NMSE
		(%)	(%)	(%)	(%)	
Full PCGM-Net-DA	Protect identity	83.39 ± 1.64	8.92 ± 1.46	99.50 ± 0.36	42.61 ± 6.46	0.102 ± 0.025
Full PCGM-Net-DA	Protect location	77.55 ± 2.91	80.19 ± 5.06	0.38 ± 0.12	49.55 ± 4.39	0.301 ± 0.095
Without policy conditioning ^a^	Any request	82.40 ± 0.96	18.26 ± 2.47	1.33 ± 0.44	44.02 ± 4.34	0.347 ± 0.053
Without keep loss	Protect location	78.51 ± 2.29	12.10 ± 8.47	0.26 ± 0.29	45.04 ± 2.26	0.369 ± 0.117

^a^ All three requested policies produced identical outputs without policy conditioning.

**Table 8:** One-factor-at-a-time sensitivity of semantic performance under clean and moderate CSI. Values are mean ± sample standard deviation across three model-training seeds; moderate results are averaged over three degradation realizations within each model.

Configuration	Clean CSI			Moderate Degradation		
	Act. Acc.	Id. Acc.	Loc. Acc.	Act. Acc.	Id. Acc.	Loc. Acc.
	(%)	(%)	(%)	(%)	(%)	(%)
Reference	74.79 ± 0.96	6.09 ± 2.40	0.29 ± 0.14	42.51 ± 1.45	17.46 ± 2.10	3.12 ± 0.61
ϵ=1.5	73.60 ± 4.79	7.97 ± 2.97	0.35 ± 0.18	42.64 ± 1.99	22.41 ± 0.30	2.29 ± 0.54
ϵ=4.5	76.09 ± 0.25	5.30 ± 1.25	0.29 ± 0.18	43.36 ± 2.81	18.66 ± 1.03	2.61 ± 0.87
$\lambda_{\mathrm{sparse}}=0.0075$	74.79 ± 1.46	6.47 ± 0.75	0.21 ± 0.11	42.91 ± 5.73	18.52 ± 0.83	2.23 ± 0.34
$\lambda_{\mathrm{sparse}}=0.03$	77.14 ± 2.99	5.38 ± 0.25	0.28 ± 0.08	44.73 ± 6.00	18.81 ± 2.13	2.79 ± 0.95
$\lambda_{\mathrm{dist}}=0.01$	74.79 ± 1.00	6.54 ± 1.20	0.31 ± 0.00	42.62 ± 4.38	17.48 ± 1.19	2.34 ± 0.05
$\lambda_{\mathrm{dist}}=0.04$	76.44 ± 1.43	7.10 ± 2.72	0.37 ± 0.14	44.38 ± 4.96	21.86 ± 1.96	2.45 ± 1.13
Privacy scale =0.5	83.24 ± 1.33	10.97 ± 3.00	0.30 ± 0.05	52.38 ± 1.44	26.07 ± 3.21	5.55 ± 2.12
Privacy scale =1.5	70.06 ± 3.58	4.44 ± 1.43	0.13 ± 0.05	39.57 ± 4.12	16.00 ± 0.65	2.56 ± 1.53

**Table 9:** Comparison with learned full-map protect-both baselines. Values are mean ± sample standard deviation across three independently trained models under the same test-set-isolated protocol.

Condition	Method	Act. Acc.	Act. F1	Id. Acc.	Id. F1	Loc. Acc.	Loc. F1	NMSE
		(%)	(%)	(%)	(%)	(%)	(%)	
Clean	PCGM-Net-DA	74.79 ± 0.96	74.72 ± 1.00	6.09 ± 2.40	5.66 ± 2.05	0.29 ± 0.14	0.43 ± 0.28	0.402 ± 0.150
Clean	GRL autoencoder	13.90 ± 0.45	8.79 ± 1.68	10.10 ± 1.12	4.32 ± 0.65	2.72 ± 3.29	1.32 ± 0.96	4.337 ± 0.973
Clean	Entropy autoencoder	90.06 ± 1.98	90.09 ± 2.00	15.57 ± 3.36	14.69 ± 2.98	12.81 ± 1.62	11.65 ± 1.04	0.321 ± 0.031
Moderate	PCGM-Net-DA	42.51 ± 1.45	42.19 ± 1.41	17.46 ± 2.10	15.31 ± 1.82	3.12 ± 0.61	4.39 ± 0.74	0.368 ± 0.155
Moderate	GRL autoencoder	13.84 ± 0.05	8.45 ± 0.77	10.37 ± 0.51	4.95 ± 0.50	4.99 ± 5.61	2.51 ± 1.61	4.411 ± 0.973
Moderate	Entropy autoencoder	66.01 ± 5.10	65.83 ± 5.30	17.65 ± 1.05	16.84 ± 0.56	23.39 ± 1.20	22.72 ± 1.02	0.288 ± 0.027

**Table 10:** Representation-level structural diagnostics for the protect-both policy. Clean values are mean ± sample standard deviation across three independently trained PCGM-Net-DA models. For moderate CSI, each model was first averaged over degradation seeds 42, 43, and 44; values are then mean ± sample standard deviation across the three model-training seeds. The temporal and subcarrier correlations compare the corresponding profiles of the input and released representation. These diagnostics do not establish physical channel equivalence.

Condition	NMSE	Perturbation Power (dB)	Temporal Profile Corr.	Subcarrier Profile Corr.	Temporal-Spectrum Cosine	Temporal-Spectrum JS
Clean	0.402 ± 0.150	−4.57 ± 1.50	0.039 ± 0.026	0.788 ± 0.013	0.724 ± 0.112	0.110 ± 0.035
Moderate	0.368 ± 0.155	−4.89 ± 1.79	0.113 ± 0.095	0.787 ± 0.014	0.861 ± 0.041	0.041 ± 0.014

**Table 11:** Clean-CSI private-semantic recoverability after supervised post-protection retraining. Each single-task attacker was trained from scratch on labeled protect-both outputs generated by protector seed 42. Values are mean ± sample standard deviation across three attacker-training seeds. Same-protector testing uses seed 42; cross-protector testing uses independently trained protectors 123 and 2026.

Architecture	Private Task	Protector Relation	Test Protector	Accuracy (%)	Macro-F1 (%)
Residual CNN	Identity	Same	42	99.87 ± 0.01	99.87 ± 0.01
Residual CNN	Identity	Cross	123	20.64 ± 9.00	17.75 ± 10.75
Residual CNN	Identity	Cross	2026	18.17 ± 4.67	13.63 ± 4.54
Residual CNN	Location	Same	42	100.00 ± 0.00	100.00 ± 0.00
Residual CNN	Location	Cross	123	47.58 ± 16.79	40.17 ± 20.82
Residual CNN	Location	Cross	2026	41.03 ± 20.99	33.33 ± 24.16
Temporal BiGRU	Identity	Same	42	98.79 ± 0.11	98.78 ± 0.11
Temporal BiGRU	Identity	Cross	123	39.10 ± 1.08	38.65 ± 1.19
Temporal BiGRU	Identity	Cross	2026	39.31 ± 2.26	38.77 ± 2.85
Temporal BiGRU	Location	Same	42	100.00 ± 0.00	100.00 ± 0.00
Temporal BiGRU	Location	Cross	123	75.09 ± 4.67	72.21 ± 5.06
Temporal BiGRU	Location	Cross	2026	74.72 ± 2.88	72.35 ± 3.65

**Table 12:** Clean-CSI activity recognition by an independent temporal BiGRU that was not used during PCGM-Net optimization. Protected-domain evaluation uses the same protector (seed 42) used to generate the protected or mixed training data. Values are mean ± sample standard deviation across three activity-model-training seeds.

Training Domain	Test Domain	Activity Accuracy (%)	Activity Macro-F1 (%)
Raw	Raw	92.98 ± 0.91	92.98 ± 0.91
Raw	Protected	36.04 ± 1.53	35.07 ± 1.83
Protected	Raw	42.51 ± 0.55	42.56 ± 0.89
Protected	Protected	94.09 ± 1.16	94.09 ± 1.16
Mixed	Raw	89.83 ± 1.07	89.83 ± 1.03
Mixed	Protected	89.76 ± 0.29	89.78 ± 0.30

**Table 13:** Model complexity and batch-size-1 inference performance on an NVIDIA GeForce RTX 5070 Ti Laptop GPU with 12 GB of dedicated memory. Latency was measured after warm-up using 100 timed iterations. Approximate FLOPs are reported as twice the multiply–accumulate count for Conv2d and Linear operations; normalization, activation, and element-wise gated-composition operations are not included.

Component	Params	Approx. FLOPs per Sample	Size	Mean Latency	P95 Latency	Throughput	Peak GPU Memory
		(M)	(MiB)	(ms)	(ms)	(Samples s^−1^)	(MiB)
Semantic classifier	260,439	66.32	1.00	0.72	0.79	1393	11.73
PCGM generator	102,946	368.29	0.39	0.83	0.88	1210	12.87
Full pipeline	363,385	434.62	1.39	2.18	2.26	458	12.87

## Data Availability

The raw dataset is not currently deposited in a public repository because it contains human-sensing records associated with anonymized identity labels and room-based location labels, and unrestricted public release was not covered by the original data-collection arrangements. Processed CSI tensors, fixed train–validation split indices, label mappings, configuration files, and evaluation code are available from the corresponding authors upon reasonable request, subject to institutional approval and participant-privacy restrictions.

## Supplementary Materials

The following supporting information can be downloaded at https://www.mdpi.com/article/10.3390/s26165096/s1, Table S1: Reference PCGM-Net-DA optimization coefficients. Values were selected using training and validation data only and fixed before test evaluation; Table S2: Synthetic CSI degradation levels. The five operators are additive Gaussian noise, random gain shift, point-wise dropout, temporal dropout, and subcarrier dropout. Dropped entries were replaced by the normalized training-set mean. Severe degradation was excluded from evaluator and generator training and from checkpoint selection; Table S3: Principal architecture and degradation-training ablations under moderate CSI and the protect-both policy. Each trained model was first averaged over degradation seeds 42, 43, and 44; values are then mean ± sample standard deviation across three model-training seeds; Table S4: Complete policy-selectivity diagnosis under clean CSI. Values are mean ± sample standard deviation across three independently trained models; Table S5: Complete one-factor-at-a-time sensitivity results under clean CSI. Values are mean ± sample standard deviation across three independently trained models; Table S6: Complete one-factor-at-a-time sensitivity results under moderate CSI. Each trained model was first averaged over degradation seeds 42, 43, and 44; values are then mean ± sample standard deviation across model-training seeds; Table S7: Complete structural and spectral diagnostics across all policies. NMSE and maximum absolute residual were computed after inverse normalization; the residual bound ϵ=3.0 applies in normalized generator space. Temporal and subcarrier correlations compare the corresponding aggregate profiles of the generator input and released representation, whereas the spectral cosine and Jensen–Shannon (JS) statistics compare their temporal spectra. For moderate CSI, each model was first averaged over degradation seeds 42, 43, and 44. Values are mean ± sample standard deviation across three PCGM-Net-DA model seeds; Table S8: Mask concentration and adjacent-position diagnostics across all policies. The top-25% energy fraction reports the proportion of the implemented mask-energy statistic assigned to the highest-intensity quartile within each sample. Temporal and subcarrier lag-1 differences summarize adjacent-position variation along the indicated axis. These descriptive diagnostics were not used for model fitting. For moderate CSI, each model was first averaged over degradation seeds 42, 43, and 44; values are then mean ± sample standard deviation across three PCGM-Net-DA model seeds; Table S9: Post-protection attacker performance for the residual CNN. For degraded conditions, each attacker was first averaged over final-test degradation seeds 42, 43, and 44; values are then mean ± sample standard deviation across three attacker-training seeds. Clean results are mean ± sample standard deviation across the same attacker-training seeds; Table S10: Post-protection attacker performance for the temporal BiGRU. For degraded conditions, each attacker was first averaged over final-test degradation seeds 42, 43, and 44; values are then mean ± sample standard deviation across three attacker-training seeds. Clean results are mean ± sample standard deviation across the same attacker-training seeds; Table S11: Uniform logit-averaged residual CNN attacker ensembles. Clean evaluation is deterministic; degraded values are mean ± standard deviation across final-test degradation seeds 42, 43, and 44; Table S12: Uniform logit-averaged temporal BiGRU attacker ensembles. Clean evaluation is deterministic; degraded values are mean ± standard deviation across final-test degradation seeds 42, 43, and 44; Table S13: Complete independent activity-model results across raw, reference-protector, and independent-protector domains. For degraded conditions, each activity model was first averaged over final-test degradation seeds 42, 43, and 44; values are then mean ± sample standard deviation across three activity-model seeds. Clean results are mean ± sample standard deviation across the same activity-model seeds. For models trained only on raw CSI, “Same” denotes testing on protected data generated by the reference protector (seed 42), whereas “Cross” denotes testing on independently trained protectors (seeds 123 and 2026); the raw-trained model itself was not exposed to any protector during training; Table S14: Complete CPU latency and throughput benchmark on the AMD Ryzen 9 8945HX host (32 logical cores). Each setting used 30 warm-up iterations and 100 timed iterations. Energy was not measured.

## Abbreviations

The following abbreviations are used in this manuscript:

AP	Access point
BiGRU	Bidirectional gated recurrent unit
CE	Cross-entropy
CNN	Convolutional neural network
CPU	Central processing unit
CSI	Channel state information
FLOPs	Floating-point operations
FNR	False-negative rate
GRL	Gradient reversal layer
GPU	Graphics processing unit
IIoT	Industrial Internet of Things
ISAC	Integrated sensing and communication
JS	Jensen–Shannon
NMSE	Normalized mean-square error
OFDM	Orthogonal frequency-division multiplexing
P95	95th-percentile latency
PCGM-Net	Policy-conditioned generative masking network
PCGM-Net-DA	Degradation-aware PCGM-Net
ReLU	Rectified linear unit
STA	Station
