# Supplementary material for: PCGM-Net: Policy-Conditioned Local Generative Masking for Privacy-Preserving Wi-Fi CSI Sensing
Source: Sensors (Basel). 2026 Aug 11;26(16):5096. doi: 10.3390/s26165096 (PMC13517445; doi:10.3390/s26165096)
Supplement: Supplementary file 1 [file sensors-26-05096-s001.zip › sensors-4430574-supplementary.pdf]

Supporting Information

PCGM-Net: Policy-Conditioned Local Generative Masking for Privacy-Preserving Wi-Fi CSI Sensing

Wei Zhang, Yifu Zeng, Qinglong Tian, Qingmiao Xiong, Honglei Chai, Yingchun Yan, and Yuxi Xiao

College of Computer Science and Engineering, Changsha University, Changsha 410022, China

Correspondence: zengyifu@hnu.edu.cn (Y.Z.); zhangw@ccsu.edu.cn (W.Z.)

Experimental Protocols and Additional Results

The supporting tables provide the complete coefficient and degradation settings, additional moderate-degradation results for the principal architecture and degradation-training variants, complete policy-selectivity diagnostics and clean- and moderate-condition sensitivity results, structural and mask diagnostics, adaptive-attacker results, downstream-activity results, and CPU-profiling results referenced in the main manuscript. Unless otherwise stated, variability is reported as the sample standard deviation across the independently trained models or attackers specified in each caption. Severe degradation was reserved for stress testing and was not used for evaluator training, generator training, or checkpoint selection.

Table S1: Reference PCGM-Net-DA optimization coefficients. Values were selected using training and validation data only and fixed before test evaluation.

Parameter	Value	Function
ϵ	3.0	Element-wise normalized residual bound
κ	0.0	Untargeted privacy-margin offset
λ_{act}	3.0	Authorized activity preservation
λ_{sem}	1.0	Joint private-semantic scale
$w_{\text{priv}}^{\text{id}}$	2.0	Identity suppression
$w_{\text{priv}}^{\text{loc}}$	8.0	Location suppression
$w_{\text{keep}}^{\text{id}}, w_{\text{keep}}^{\text{loc}}$	1.0, 1.0	Unselected-semantic retention
λ_{sparse}	0.015	Mean mask regularization
λ_{dist}	0.02	Released-view distortion regularization

Table S2: Synthetic CSI degradation levels. The five operators are additive Gaussian noise, random gain shift, point-wise dropout, temporal dropout, and subcarrier dropout. Dropped entries were replaced by the normalized training-set mean. Severe degradation was excluded from evaluator and generator training and from checkpoint selection.

Condition	Noise ratio	Gain std.	Point dropout	Time dropout	Subcarrier dropout
Clean	0.00	0.00	0.00	0.00	0.00
Mild	0.03	0.03	0.03	0.05	0.05
Moderate	0.08	0.08	0.08	0.10	0.10
Severe	0.15	0.15	0.15	0.20	0.20

During evaluator and PCGM-Net-DA training, one of the clean, mild, and moderate conditions was sampled uniformly for each mini-batch, and a stochastic realization was generated within the selected condition. Validation checkpoint selection used degradation seeds 101, 102, and 103, whereas final test evaluation used degradation seeds 42, 43, and 44.

Table S3: Principal architecture and degradation-training ablations under moderate CSI and the protect-both policy. Each trained model was first averaged over degradation seeds 42, 43, and 44; values are then mean ± sample standard deviation across three model-training seeds.

Variant	Act. Acc. (%)	Act. F1 (%)	Id. Acc. (%)	Loc. Acc. (%)	Mask intensity (%)	NMSE
Full PCGM-Net-DA	42.51 ± 1.45	42.19 ± 1.41	17.46 ± 2.10	3.12 ± 0.61	51.31 ± 3.52	0.3677 ± 0.1548
w/o policy conditioning	44.71 ± 1.43	44.29 ± 1.41	27.14 ± 2.90	16.54 ± 2.11	43.78 ± 4.14	0.3013 ± 0.0637
Mask branch only	29.89 ± 2.03	29.28 ± 2.29	17.99 ± 0.42	8.86 ± 0.75	54.21 ± 1.27	0.1882 ± 0.0214
Residual-patch branch only	49.67 ± 3.98	49.59 ± 4.07	15.50 ± 1.94	2.14 ± 0.33	100.00 ± 0.00	0.4899 ± 0.0757
w/o degradation-aware training	24.18 ± 4.14	22.15 ± 3.93	17.00 ± 5.20	27.44 ± 8.35	48.43 ± 0.36	0.3109 ± 0.1728

Table S4: Complete policy-selectivity diagnosis under clean CSI. Values are mean ± sample standard deviation across three independently trained models.

Variant	Policy	Act. Acc. (%)	Id. Acc. (%)	Loc. Acc. (%)	Mask intensity (%)	NMSE
Full PCGM-Net-DA	Protect identity	83.39 ± 1.64	8.92 ± 1.46	99.50 ± 0.36	42.61 ± 6.46	0.1021 ± 0.0251
Full PCGM-Net-DA	Protect location	77.55 ± 2.91	80.19 ± 5.06	0.38 ± 0.12	49.55 ± 4.39	0.3009 ± 0.0954
Full PCGM-Net-DA	Protect both	74.79 ± 0.96	6.09 ± 2.40	0.29 ± 0.14	51.19 ± 3.70	0.4020 ± 0.1497
w/o policy conditioning	Protect identity	82.40 ± 0.96	18.26 ± 2.47	1.33 ± 0.44	44.02 ± 4.34	0.3474 ± 0.0526
w/o policy conditioning	Protect location	82.40 ± 0.96	18.26 ± 2.47	1.33 ± 0.44	44.02 ± 4.34	0.3474 ± 0.0526
w/o policy conditioning	Protect both	82.40 ± 0.96	18.26 ± 2.47	1.33 ± 0.44	44.02 ± 4.34	0.3474 ± 0.0526
w/o keep loss	Protect identity	84.94 ± 1.04	7.87 ± 2.83	93.40 ± 8.59	31.05 ± 1.61	0.1328 ± 0.0074
w/o keep loss	Protect location	78.51 ± 2.29	12.10 ± 8.47	0.26 ± 0.29	45.04 ± 2.26	0.3691 ± 0.1173
w/o keep loss	Protect both	76.53 ± 2.43	4.33 ± 0.92	0.18 ± 0.17	45.41 ± 2.09	0.3790 ± 0.1165

Table S5: Complete one-factor-at-a-time sensitivity results under clean CSI. Values are mean ± sample standard deviation across three independently trained models.

Configuration	Act. Acc. (%)	Act. F1 (%)	Id. Acc. (%)	Loc. Acc. (%)	Mask intensity (%)	NMSE
Reference	74.79 ± 0.96	74.72 ± 1.00	6.09 ± 2.40	0.29 ± 0.14	51.19 ± 3.70	0.402 ± 0.150
$\epsilon = 1.5$	73.60 ± 4.79	73.57 ± 4.81	7.97 ± 2.97	0.35 ± 0.18	67.09 ± 2.80	0.297 ± 0.052
$\epsilon = 4.5$	76.09 ± 0.25	76.06 ± 0.26	5.30 ± 1.25	0.29 ± 0.18	37.03 ± 7.41	0.389 ± 0.140
$\lambda_{\text{sparse}} = 0.0075$	74.79 ± 1.46	74.82 ± 1.45	6.47 ± 0.75	0.21 ± 0.11	47.80 ± 3.19	0.390 ± 0.143
$\lambda_{\text{sparse}} = 0.03$	77.14 ± 2.99	77.11 ± 2.98	5.38 ± 0.25	0.28 ± 0.08	46.11 ± 4.28	0.350 ± 0.093
$\lambda_{\text{dist}} = 0.01$	74.79 ± 1.00	74.75 ± 0.96	6.54 ± 1.20	0.31 ± 0.00	48.27 ± 4.49	0.391 ± 0.126
$\lambda_{\text{dist}} = 0.04$	76.44 ± 1.43	76.48 ± 1.35	7.10 ± 2.72	0.37 ± 0.14	43.58 ± 0.44	0.296 ± 0.062
Privacy scale = 0.5	83.24 ± 1.33	83.23 ± 1.35	10.97 ± 3.00	0.30 ± 0.05	46.47 ± 4.20	0.311 ± 0.097
Privacy scale = 1.5	70.06 ± 3.58	69.96 ± 3.70	4.44 ± 1.43	0.13 ± 0.05	46.20 ± 3.89	0.320 ± 0.065

Table S6: Complete one-factor-at-a-time sensitivity results under moderate CSI. Each trained model was first averaged over degradation seeds 42, 43, and 44; values are then mean ± sample standard deviation across model-training seeds.

Configuration	Act. Acc. (%)	Act. F1 (%)	Id. Acc. (%)	Loc. Acc. (%)	Mask intensity (%)	NMSE
Reference	42.51 ± 1.45	42.19 ± 1.41	17.46 ± 2.10	3.12 ± 0.61	51.31 ± 3.52	0.368 ± 0.155
$\epsilon = 1.5$	42.64 ± 1.99	41.97 ± 2.07	22.41 ± 0.30	2.29 ± 0.54	65.76 ± 2.31	0.255 ± 0.033
$\epsilon = 4.5$	43.36 ± 2.81	42.81 ± 3.00	18.66 ± 1.03	2.61 ± 0.87	37.31 ± 7.07	0.325 ± 0.123
$\lambda_{\text{sparse}} = 0.0075$	42.91 ± 5.73	42.50 ± 5.88	18.52 ± 0.83	2.23 ± 0.34	48.12 ± 3.13	0.340 ± 0.142
$\lambda_{\text{sparse}} = 0.03$	44.73 ± 6.00	44.39 ± 6.18	18.81 ± 2.13	2.79 ± 0.95	47.08 ± 4.02	0.320 ± 0.089
$\lambda_{\text{dist}} = 0.01$	42.62 ± 4.38	42.21 ± 4.61	17.48 ± 1.19	2.34 ± 0.05	48.63 ± 4.13	0.354 ± 0.124
$\lambda_{\text{dist}} = 0.04$	44.38 ± 4.96	43.91 ± 5.21	21.86 ± 1.96	2.45 ± 1.13	43.57 ± 0.95	0.259 ± 0.059
Privacy scale = 0.5	52.38 ± 1.44	52.08 ± 1.45	26.07 ± 3.21	5.55 ± 2.12	45.86 ± 3.78	0.275 ± 0.095
Privacy scale = 1.5	39.57 ± 4.12	39.10 ± 4.22	16.00 ± 0.65	2.56 ± 1.53	46.30 ± 3.71	0.277 ± 0.054

Table S7: Complete structural and spectral diagnostics across all policies. NMSE and maximum absolute residual were computed after inverse normalization; the residual bound $\epsilon = 3.0$ applies in normalized generator space. Temporal and subcarrier correlations compare the corresponding aggregate profiles of the generator input and released representation, whereas the spectral cosine and Jensen–Shannon (JS) statistics compare their temporal spectra. For moderate CSI, each model was first averaged over degradation seeds 42, 43, and 44. Values are mean ± sample standard deviation across three PCGM-Net-DA model seeds.

Policy	Condition	NMSE	Perturb. power (dB)	Temporal corr.	Subcarrier corr.	Spectral cosine	Spectral JS	Max. residual
Protect identity	Clean	0.102 ± 0.025	-10.26 ± 1.00	0.164 ± 0.085	0.930 ± 0.013	0.827 ± 0.068	0.072 ± 0.027	324.9 ± 52.0
Protect identity	Moderate	0.093 ± 0.035	-10.72 ± 1.54	0.359 ± 0.157	0.927 ± 0.016	0.948 ± 0.015	0.015 ± 0.004	319.7 ± 57.7
Protect location	Clean	0.301 ± 0.095	-5.75 ± 1.19	0.058 ± 0.031	0.830 ± 0.007	0.741 ± 0.107	0.106 ± 0.031	402.9 ± 28.9
Protect location	Moderate	0.257 ± 0.085	-6.33 ± 1.34	0.160 ± 0.103	0.833 ± 0.014	0.864 ± 0.037	0.039 ± 0.012	392.5 ± 36.7
Protect both	Clean	0.402 ± 0.150	-4.57 ± 1.50	0.039 ± 0.026	0.788 ± 0.013	0.724 ± 0.112	0.110 ± 0.035	409.6 ± 33.6
Protect both	Moderate	0.368 ± 0.155	-4.89 ± 1.79	0.113 ± 0.095	0.787 ± 0.014	0.861 ± 0.041	0.041 ± 0.014	403.6 ± 43.0

Table S8: Mask concentration and adjacent-position diagnostics across all policies. The top-25% energy fraction reports the proportion of the implemented mask-energy statistic assigned to the highest-intensity quartile within each sample. Temporal and subcarrier lag-1 differences summarize adjacent-position variation along the indicated axis. These descriptive diagnostics were not used for model fitting. For moderate CSI, each model was first averaged over degradation seeds 42, 43, and 44; values are then mean ± sample standard deviation across three PCGM-Net-DA model seeds.

Policy	Condition	Mask intensity (%)	Top-25% energy (%)	Temporal lag-1 difference	Subcarrier lag-1 difference
Protect identity	Clean	42.61 ± 6.46	37.83 ± 4.35	0.404 ± 0.035	0.109 ± 0.010
Protect identity	Moderate	43.35 ± 8.22	37.06 ± 5.51	0.370 ± 0.090	0.064 ± 0.015
Protect location	Clean	49.55 ± 4.39	36.37 ± 2.46	0.621 ± 0.201	0.399 ± 0.090
Protect location	Moderate	49.70 ± 4.60	35.54 ± 2.46	0.480 ± 0.171	0.375 ± 0.080
Protect both	Clean	51.19 ± 3.70	36.43 ± 2.83	0.607 ± 0.163	0.443 ± 0.105
Protect both	Moderate	51.31 ± 3.52	35.66 ± 2.73	0.476 ± 0.230	0.407 ± 0.108

Table S9: Post-protection attacker performance for the residual CNN. For degraded conditions, each attacker was first averaged over final-test degradation seeds 42, 43, and 44; values are then mean ± sample standard deviation across three attacker-training seeds. Clean results are mean ± sample standard deviation across the same attacker-training seeds.

Private task	Protector relation	Test protector	Condition	Accuracy (%)	Macro-F1 (%)
Identity	Same	42	Clean	99.87 ± 0.01	99.87 ± 0.01
Identity	Same	42	Moderate	90.86 ± 0.27	90.86 ± 0.29
Identity	Same	42	Severe	64.09 ± 0.41	64.08 ± 0.41
Identity	Cross	123	Clean	20.64 ± 9.00	17.75 ± 10.75
Identity	Cross	123	Moderate	20.31 ± 7.83	17.06 ± 9.96
Identity	Cross	123	Severe	16.79 ± 5.32	12.40 ± 7.51
Identity	Cross	2026	Clean	18.17 ± 4.67	13.63 ± 4.54
Identity	Cross	2026	Moderate	14.69 ± 3.19	8.89 ± 3.89
Identity	Cross	2026	Severe	12.70 ± 1.92	6.81 ± 3.37
Location	Same	42	Clean	100.00 ± 0.00	100.00 ± 0.00
Location	Same	42	Moderate	99.99 ± 0.01	99.99 ± 0.01
Location	Same	42	Severe	98.28 ± 0.32	98.28 ± 0.32
Location	Cross	123	Clean	47.58 ± 16.79	40.17 ± 20.82
Location	Cross	123	Moderate	37.49 ± 12.41	29.86 ± 14.38
Location	Cross	123	Severe	31.43 ± 10.39	23.24 ± 12.11
Location	Cross	2026	Clean	41.03 ± 20.99	33.33 ± 24.16
Location	Cross	2026	Moderate	37.21 ± 17.54	28.64 ± 20.16
Location	Cross	2026	Severe	30.98 ± 12.43	22.17 ± 14.95

Table S10: Post-protection attacker performance for the temporal BiGRU. For degraded conditions, each attacker was first averaged over final-test degradation seeds 42, 43, and 44; values are then mean ± sample standard deviation across three attacker-training seeds. Clean results are mean ± sample standard deviation across the same attacker-training seeds.

Private task	Protector relation	Test protector	Condition	Accuracy (%)	Macro-F1 (%)
Identity	Same	42	Clean	98.79 ± 0.11	98.78 ± 0.11
Identity	Same	42	Moderate	88.50 ± 0.21	88.49 ± 0.20
Identity	Same	42	Severe	70.48 ± 0.17	70.50 ± 0.14
Identity	Cross	123	Clean	39.10 ± 1.08	38.65 ± 1.19
Identity	Cross	123	Moderate	36.99 ± 2.75	36.49 ± 2.85
Identity	Cross	123	Severe	32.46 ± 1.88	31.50 ± 2.29
Identity	Cross	2026	Clean	39.31 ± 2.26	38.77 ± 2.85
Identity	Cross	2026	Moderate	36.51 ± 2.01	35.99 ± 2.25
Identity	Cross	2026	Severe	31.42 ± 1.08	30.97 ± 1.10
Location	Same	42	Clean	100.00 ± 0.00	100.00 ± 0.00
Location	Same	42	Moderate	99.99 ± 0.00	99.99 ± 0.00
Location	Same	42	Severe	99.22 ± 0.05	99.22 ± 0.05
Location	Cross	123	Clean	75.09 ± 4.67	72.21 ± 5.06
Location	Cross	123	Moderate	73.30 ± 2.58	70.87 ± 3.79
Location	Cross	123	Severe	68.78 ± 0.95	65.78 ± 2.16
Location	Cross	2026	Clean	74.72 ± 2.88	72.35 ± 3.65
Location	Cross	2026	Moderate	73.68 ± 3.02	72.18 ± 3.44
Location	Cross	2026	Severe	70.21 ± 3.63	68.94 ± 3.66

Table S11: Uniform logit-averaged residual CNN attacker ensembles. Clean evaluation is deterministic; degraded values are mean ± standard deviation across final-test degradation seeds 42, 43, and 44.

Private task	Protector relation	Test protector	Condition	Accuracy (%)	Macro-F1 (%)
Identity	Same	42	Clean	99.83	99.83
Identity	Same	42	Moderate	92.80 ± 0.17	92.81 ± 0.16
Identity	Same	42	Severe	70.06 ± 0.47	70.05 ± 0.48
Identity	Cross	123	Clean	22.76	22.47
Identity	Cross	123	Moderate	17.62 ± 0.46	14.09 ± 0.53
Identity	Cross	123	Severe	13.70 ± 0.24	8.38 ± 0.53
Identity	Cross	2026	Clean	15.24	11.93
Identity	Cross	2026	Moderate	12.97 ± 0.20	7.05 ± 0.27
Identity	Cross	2026	Severe	11.81 ± 0.26	4.62 ± 0.20
Location	Same	42	Clean	100.00	100.00
Location	Same	42	Moderate	100.00 ± 0.00	100.00 ± 0.00
Location	Same	42	Severe	98.87 ± 0.24	98.87 ± 0.24
Location	Cross	123	Clean	48.43	41.32
Location	Cross	123	Moderate	37.58 ± 0.18	26.39 ± 0.33
Location	Cross	123	Severe	32.61 ± 0.28	22.11 ± 0.36
Location	Cross	2026	Clean	41.02	35.67
Location	Cross	2026	Moderate	35.02 ± 0.29	28.73 ± 0.25
Location	Cross	2026	Severe	29.05 ± 0.33	22.45 ± 0.53

Table S12: Uniform logit-averaged temporal BiGRU attacker ensembles. Clean evaluation is deterministic; degraded values are mean ± standard deviation across final-test degradation seeds 42, 43, and 44.

Private task	Protector relation	Test protector	Condition	Accuracy (%)	Macro-F1 (%)
Identity	Same	42	Clean	98.90	98.90
Identity	Same	42	Moderate	90.50 ± 0.10	90.49 ± 0.10
Identity	Same	42	Severe	75.63 ± 0.88	75.65 ± 0.87
Identity	Cross	123	Clean	47.07	47.37
Identity	Cross	123	Moderate	43.06 ± 0.35	43.21 ± 0.33
Identity	Cross	123	Severe	37.52 ± 1.04	36.99 ± 1.14
Identity	Cross	2026	Clean	47.31	48.03
Identity	Cross	2026	Moderate	43.03 ± 0.18	43.31 ± 0.19
Identity	Cross	2026	Severe	36.94 ± 0.56	37.06 ± 0.58
Location	Same	42	Clean	100.00	100.00
Location	Same	42	Moderate	99.99 ± 0.01	99.99 ± 0.01
Location	Same	42	Severe	99.44 ± 0.23	99.44 ± 0.22
Location	Cross	123	Clean	77.05	74.32
Location	Cross	123	Moderate	75.71 ± 0.31	73.30 ± 0.56
Location	Cross	123	Severe	72.83 ± 0.30	69.75 ± 0.19
Location	Cross	2026	Clean	76.64	74.50
Location	Cross	2026	Moderate	77.37 ± 0.47	76.03 ± 0.48
Location	Cross	2026	Severe	75.55 ± 0.08	74.23 ± 0.10

Table S13: Complete independent activity-model results across raw, reference-protector, and independent-protector domains. For degraded conditions, each activity model was first averaged over final-test degradation seeds 42, 43, and 44; values are then mean ± sample standard deviation across three activity-model seeds. Clean results are mean ± sample standard deviation across the same activity-model seeds. For models trained only on raw CSI, "Same" denotes testing on protected data generated by the reference protector (seed 42), whereas "Cross" denotes testing on independently trained protectors (seeds 123 and 2026); the raw-trained model itself was not exposed to any protector during training.

Training domain	Test domain	Relation	Test protector	Condition	Accuracy (%)	Macro-F1 (%)
Raw	Protected	Same	42	Clean	36.04 ± 1.53	35.07 ± 1.83
Raw	Protected	Same	42	Moderate	31.57 ± 2.11	30.19 ± 2.39
Raw	Protected	Cross	123	Clean	41.29 ± 1.61	40.58 ± 1.76
Raw	Protected	Cross	123	Moderate	38.21 ± 1.67	37.28 ± 1.85
Raw	Protected	Cross	2026	Clean	39.78 ± 2.18	39.18 ± 2.42
Raw	Protected	Cross	2026	Moderate	35.17 ± 2.11	34.37 ± 2.32
Raw	Raw	N/A	raw	Clean	92.98 ± 0.91	92.98 ± 0.91
Raw	Raw	N/A	raw	Moderate	75.68 ± 0.15	75.69 ± 0.15
Protected	Protected	Same	42	Clean	94.09 ± 1.16	94.09 ± 1.16
Protected	Protected	Same	42	Moderate	72.18 ± 0.10	72.18 ± 0.11
Protected	Protected	Cross	123	Clean	26.22 ± 1.54	24.53 ± 1.46
Protected	Protected	Cross	123	Moderate	25.47 ± 1.38	23.36 ± 1.63
Protected	Protected	Cross	2026	Clean	27.38 ± 2.30	25.57 ± 3.36
Protected	Protected	Cross	2026	Moderate	25.55 ± 1.51	23.75 ± 1.92
Protected	Raw	N/A	raw	Clean	42.51 ± 0.55	42.56 ± 0.89
Protected	Raw	N/A	raw	Moderate	38.38 ± 0.29	38.07 ± 0.42
Mixed	Protected	Same	42	Clean	89.76 ± 0.29	89.78 ± 0.30
Mixed	Protected	Same	42	Moderate	70.90 ± 0.13	70.88 ± 0.13
Mixed	Protected	Cross	123	Clean	41.29 ± 0.68	40.76 ± 0.23
Mixed	Protected	Cross	123	Moderate	39.18 ± 1.56	38.51 ± 1.82
Mixed	Protected	Cross	2026	Clean	40.98 ± 2.05	40.65 ± 2.58
Mixed	Protected	Cross	2026	Moderate	36.85 ± 1.99	36.27 ± 2.20
Mixed	Raw	N/A	raw	Clean	89.83 ± 1.07	89.83 ± 1.03
Mixed	Raw	N/A	raw	Moderate	72.41 ± 0.21	72.40 ± 0.21

Table S14: Complete CPU latency and throughput benchmark on the AMD Ryzen 9 8945HX host (32 logical cores). Each setting used 30 warm-up iterations and 100 timed iterations. Energy was not measured.

Threads	Batch	Component	Mean (ms)	P95 (ms)	P99 (ms)	Samples/s
1	1	Semantic evaluator	1.474	1.671	1.926	678.4
1	1	PCGM generator	2.847	2.954	3.001	351.2
1	1	Complete pipeline	4.247	4.425	4.501	235.5
1	32	Semantic evaluator	33.064	48.847	78.416	967.8
1	32	PCGM generator	100.570	106.017	107.391	318.2
1	32	Complete pipeline	127.408	132.728	136.846	251.2
1	256	Semantic evaluator	238.575	241.937	243.304	1073.0
1	256	PCGM generator	718.582	729.366	731.532	356.3
1	256	Complete pipeline	960.567	974.902	976.721	266.5
4	1	Semantic evaluator	0.569	0.615	0.686	1758.6
4	1	PCGM generator	1.268	1.345	1.371	788.5
4	1	Complete pipeline	1.967	2.060	3.362	508.4
4	32	Semantic evaluator	7.903	8.668	9.733	4049.1
4	32	PCGM generator	25.058	26.315	27.725	1277.0
4	32	Complete pipeline	32.948	34.252	34.628	971.2
4	256	Semantic evaluator	75.768	78.484	79.550	3378.7
4	256	PCGM generator	261.861	265.781	266.328	977.6
4	256	Complete pipeline	342.258	348.039	351.170	748.0
8	1	Semantic evaluator	0.404	0.419	0.433	2476.2
8	1	PCGM generator	0.994	1.019	1.045	1006.1
8	1	Complete pipeline	1.495	1.810	2.612	669.1
8	32	Semantic evaluator	4.627	6.307	6.988	6915.9
8	32	PCGM generator	14.575	16.484	18.874	2195.6
8	32	Complete pipeline	19.530	23.552	31.673	1638.5
8	256	Semantic evaluator	45.295	49.416	53.485	5651.8
8	256	PCGM generator	188.146	199.624	211.474	1360.6
8	256	Complete pipeline	234.483	243.189	250.089	1091.8
